# MiR-668-5p targets ZNRF3, an E3 ubiquitin ligase to enhance osteoblast function and alleviate senescence in doxorubicin-induced age-related bone loss

**DOI:** 10.3389/fendo.2025.1693659

**Published:** 2025-10-27

**Authors:** Devendra Pratap Singh, Alok Tripathi, Ankita Paul, Saurabh Kumar Kaushal, Megha Dixit, Divya Singh

**Affiliations:** ^1^ Division of Endocrinology, CSIR-Central Drug Research Institute, Lucknow, India; ^2^ Academy of Scientific and Innovative Research (AcSIR), Ghaziabad, India

**Keywords:** microRNA, Znrf3, bone, osteoporosis, osteoblast, aging, cellular senescence, oxidative stress

## Abstract

**Introduction:**

Aging is a complex biological phenomenon that drives the progression of numerous chronic conditions, including osteoporosis, which is characterized by bone loss and increased risk of fragility fractures. The study of microRNA (miRNA) functions has provided valuable insights into the mechanisms that regulate aging and the senescence process. In this study, we identified microRNA 668-5p (miR-668-5p) as a novel regulator of osteoblast function and further elucidated its role in age-related bone loss driven by cellular senescence.

**Methods:**

miR-668-5p function was assessed through mimic transfection in osteoblasts, with osteogenic differentiation evaluated by osteogenic gene and protein expression. Target confirmation was conducted using a dual luciferase 3′UTR reporter assay to identify Znrf3, an E3 ubiquitin ligase and negative regulator of Wnt signaling, as a direct target of miR-668-5p. Cellular senescence was induced using doxorubicin (Doxo), a well-established agent for simulating an accelerated aging phenotype. Further, the effects of miR-668-5p on senescence markers (SA-β-gal activity, p53, p21), reactive oxygen species (ROS) levels, and Znrf3 expression were examined. *In vivo*, miR-668-5p was administered to Doxo-treated mice, and trabecular bone microarchitecture was evaluated using microCT, while bone regeneration and bone strength were analyzed using calcein incorporation and mechanical testing, respectively.

**Results:**

Transfection with the miR-668-5p mimic enhanced osteoblast differentiation and function, as indicated by increased expression of osteogenic markers. Znrf3 was confirmed as a direct target, mediating the regulation of Wnt signaling. Doxo treatment suppressed miR-668-5p expression, increased Znrf3 levels, and promoted cellular senescence and ROS. miR-668-5p augmentation mitigated these effects, reducing senescence markers and ROS production. *In vivo*, miR-668-5p administration improved trabecular bone microarchitecture, bone regeneration, and bone strength in Doxo-treated mice. Notably, Znrf3 overexpression in osteoblasts reversed the anti-senescence and pro-osteogenic effects of miR-668-5p, confirming its target-specific role.

**Discussion:**

These findings establish miR-668-5p as a key regulator of osteoblast function and bone homeostasis through its direct targeting of Znrf3 and modulation of Wnt signaling. By alleviating senescence and promoting osteogenesis, miR-668-5p demonstrates therapeutic potential in combating age-related bone loss and osteoporosis.

## 1 Introduction

Aging is one of the most significant risk factors for a variety of chronic diseases, including osteoporosis, sarcopenia, diabetes, cardiovascular disorders, and neurodegenerative conditions like Alzheimer’s disease (AD) (1, 2). Among the numerous age-associated conditions, senile osteoporosis represents a critical public health challenge due to its profound impact on skeletal integrity and its association with debilitating fractures in the elderly population. One of the critical mechanisms underpinning age-related dysfunction is cellular senescence, a state of irreversible cell cycle arrest triggered by various intrinsic and extrinsic stimuli, including DNA damage, oxidative stress, inflammation, irradiation, chemotherapeutic agents, and so forth (3, 4). In bone tissue, the accumulation of senescent osteoblasts and osteocytes impairs bone formation and accelerates age-related bone loss (5, 6).

In recent years, microRNAs (miRNAs) have emerged as critical regulators of bone remodeling, aging, and cellular senescence (7). These small and evolutionarily conserved non-coding RNAs (~22 nucleotides in length) modulate various biological processes, including cell proliferation, growth, apoptosis, regeneration, and metabolism. Importantly, several miRNAs regulate key components of osteogenic pathways, mostly through the Wnt/β-catenin signaling pathway (8), which plays a central role in bone formation, homeostasis, and aging-related processes (9, 10). The Wnt/β-catenin pathway is indispensable for maintaining bone health and has been identified as a key therapeutic target for treating bone diseases (11). Its dysregulation is also strongly implicated in aging conditions. Reduced Wnt/β-catenin signaling has been associated with cognitive decline and memory deficits in AD mouse models (10).

Our previous study investigated the microRNA signature in Phex-silenced osteoblasts (12). Phosphate-regulating endopeptidase X linked (PHEX) is an endopeptidase predominantly expressed in mature osteoblasts, osteocytes, and odontoblasts, playing a critical role in matrix mineralization. It has been extensively studied in conditions like X-linked hypophosphatemia (XLH) (13, 14). Genome-wide RNAi screens have identified the Phex gene as one of the positive regulators of the Wnt/β-catenin signaling pathway (15).

In this study, we identified the differential expression of several miRNA candidates exhibiting upregulation and downregulation in Phex-silenced osteoblasts. Notably, miR-668-5p, a novel miRNA, demonstrated a significant decrease (over threefold) in expression among the most significantly downregulated miRNAs in this analysis. In previously reported literature, the isoform miR-668-3p has been shown to protect mitochondrial dynamics in ischemic acute kidney injury (AKI) (16), regulate cardiomyocyte survival in ischemia-reperfusion injury (IRI) (17), and osteoblast progression in osteonecrosis of the femoral head (18). In another study, miR-668-3p was also identified as a potential therapeutic target for AD, an aging-related neurodegenerative disorder, by regulating oxidative stress (19). However, the functions of miR-668-5p remain unexplored, especially in bone homeostasis. Moreover, miRDB and TargetScan analysis revealed that miR-668-5p directly targets Zinc and Ring finger 3 (Znrf3), an E3 ubiquitin ligase, which is a negative regulator of the Wnt signaling pathway (20). These observations prompted us to study the role of miR-668-5p in osteogenesis and, more importantly, its potential impact on age-related bone loss driven by cellular senescence and oxidative stress. To mimic the aging condition, doxorubicin (Doxo), a common chemotherapeutic anthracycline, was used, which is well documented to induce cellular senescence and oxidative stress in various cell types (21–23). In this study, we report the protective role of miR-668-5p in suppressing aging-induced bone loss by targeting Znrf3, thereby upregulating Wnt signaling.

## 2 Materials and methods

Details of cell culture (MCO culture), immunofluorescence, measurement of mitochondrial ROS, cell apoptosis assay using annexin V-FITC/PI staining, and body composition analysis are provided in **Supplementary Information**.

### 2.1 Cell culture and miRNA transfection

Mouse calvarial osteoblasts (MCOs) were harvested from the calvariae of neonatal mouse pups (1–2 days old), following standard previously published protocols of sequential digestion (24). MicroRNA transfections were conducted using Lipofectamine RNAiMAX (Invitrogen, Carlsbad, CA, USA) reagent in Opti-MEM (Gibco, Thermo Scientific, Waltham, MA, USA) reduced serum medium following the manufacturer’s protocol. When the cells reached 50%–70% confluence, they were transfected with synthetic RNA oligonucleotides (Ambion, Thermo Scientific, Waltham, MA, USA) corresponding to mature sequences that simulate endogenous miRNAs, including miR-668-5p (mimic), antimiR-668-5p (inhibitor), and an miRNA negative control (miC) at a concentration of 50 nM using Lipofectamine RNAiMAX for a 6h duration. After the 6h transfection period, the medium was replaced with osteoblast differentiation medium. Cells were collected for subsequent analysis 48h post-miRNA transfection.

For induction of cellular senescence *in vitro*, after the transfection period, the medium was replaced with osteoblast differential medium containing 25 nM doxorubicin (Doxo) (Cayman Chemical, Ann Arbor, MI, USA) for 48h. The cultures were maintained for another 24h–36h in Doxo-free medium before collection for subsequent analysis (22, 25, 26). To validate the regulatory effect of miRNA target Znrf3 on Doxo-induced cellular senescence, osteoblast cells were transfected with miR-668-5p mimic in the presence or absence of 0.5 μg/ml recombinant Znrf3 (rZnrf3) (8328-RF- R&D systems, Minneapolis, MN, USA) in Doxo conditions.

### 2.2 Alkaline phosphatase assay and staining

ALP (alkaline phosphatase) activity was assessed following established protocols (24). Cells were seeded in a 96-well format, and post-transfection, the cells were incubated for 48h in osteoblast differentiation medium. After the incubation period, cells were washed with 1× PBS, and plates were kept at −20°C (2h–6 days). Later, plates were kept directly at 37 °C (from −20 °C) to expose the ectoenzyme Alp, and total ALP activity was measured using p-nitrophenyl phosphate (pNPP) (Sigma, St. Louis, MO, USA) as substrate. Absorbance was taken at 405 nm using a Spectra Max microplate reader (Molecular Devices San Jose, CA, USA) to quantify the enzyme activity. MCOs were seeded in 12-well plates, followed by transfection with control (miC), miR-668-5p mimic, and antimiR-668-5p. For ALP staining, the treated cell cultures were maintained for 7 days in osteoblast differential medium. At the end of the culture period, the cells were washed once with 1× PBS and subsequently fixed using 4% paraformaldehyde at room temperature for 15 min. The fixed cells were then incubated with an alkaline phosphatase substrate solution containing a chromogenic dye NBT/BCIP (Thermo Scientific, Waltham, MA, USA) for about 20–30 min at room temperature (27). After the color development, imaging was carried out using a bright-field microscope (4×).

### 2.3 Mineralization assay

After miRNA transfection, the osteoblast culture was maintained for 18 days in osteoblast differential medium with medium replenishment every 48h and repeated miRNA transfection on the 7th and 14th days. For *ex-vivo* mineralization analysis, femoral bone marrow samples from autopsied mice from various experimental groups were cultured in bone marrow culture (BMC) media containing α-MEM medium supplemented with 5% FBS, 10^−7^ M dexamethasone (Sigma, St. Louis, MO, USA), 50 μg/ml ascorbic acid (Sigma, St. Louis, MO, USA), and 10 mM β-glycerophosphate (Sigma, St. Louis, MO, USA) for 21 days. For mineralization assessment, cells were washed with 1× PBS and then fixed for 20–30 min at RT using 4% Paraformaldehyde (PFA). Alizarin Red Staining (ARS) (Sigma, St. Louis, MO, USA) was followed as described (28). In brief, cells were stained with Alizarin Red Stain (40 mM, pH 4.2) for 30–60 min at RT with mild shaking to detect calcium deposits, followed by imaging under a bright field microscope (4×). Quantification of ARS stain was done using 10% cetylpyridinium chloride (CPC) (Sigma, St. Louis, MO, USA) to dissolve the bound dye, and absorbance was measured at 595 nm.

### 2.4 qPCR analysis for miRNA and mRNA expression

miRNA isolation was performed using the mirVana miRNA isolation kit (Invitrogen, Carlsbad, CA, USA) according to the manufacturer’s instructions. The extracted miRNA was quantified via Nanodrop (Thermo Scientific, Waltham, MA, USA). Further, the TaqMan™ Advanced miRNA cDNA Synthesis Kit (Applied Biosystems, Thermo Scientific, Waltham, MA, USA) was employed for cDNA synthesis according to the manufacturer’s instructions. The synthesized and amplified cDNA was subsequently stored at −20 °C for further analysis. qPCR was conducted using the 2× TaqMan™ Fast Advanced Master mix (Applied Biosystems, Thermo Scientific, Waltham, MA, USA), 20× TaqMan™ Advanced miRNA Assay of miRNA (mmu-miR-668-5p, (Thermo Scientific, Waltham, MA, USA), and synthesized cDNA. U6snRNA (Thermo Scientific, Waltham, MA, USA) was employed as an internal control. qPCR analysis for gene (mRNA) expression studies was carried out using SYBR Green chemistry. Total RNA was isolated from treated osteoblast cells and pulverized femur bone samples using the Trizol (Invitrogen, Carlsbad, CA, USA) method, and concentration was determined using NanoDrop. One microgram of total RNA was reverse transcribed using a high-capacity cDNA Reverse Transcription Kit (Applied Biosystems, Thermo Scientific, Waltham, MA, USA), according to the manufacturer’s instructions. Quantitative mRNA expression of different genes was further carried out using PowerUp SYBR Green Master Mix (Applied Biosystems, Thermo Scientific, Waltham, MA, USA), forward and reverse primers of the respective genes, and cDNA product. All genes were examined with the StepOnePlus real-time PCR system (Applied Biosystems, Thermo Scientific, Waltham, MA, USA). The primers were designed utilizing the Primer-BLAST tool provided by NCBI, and their sequences are detailed in **Supplementary Table S1**. The relative expression levels of the target mRNA/miRNA were calculated using the log2|2—DCt|, where DCt represents the difference between the Ct value of the target mRNA/miRNA and that of the internal control Gapdh/U6snRNA.

### 2.5 miRNA target prediction and dual luciferase reporter assay

For miRNA target prediction, tools such as TargetScan (http://www.targetscan.org/vert_80/) and miRDB (http://www.mirdb.org/) were employed. For target validation, cells were transfected with 250 ng of the pEZX-MT06 vector, containing the 3′ UTR of Znrf3 (3′ UTR Znrf3 clone) (Genecopoeia, Rockville, MD, USA), and an empty pEZX-MT06 vector as the control (lacking 3′UTR). These vectors included firefly luciferase as a reporter gene under the control of the SV40 promoter and renilla luciferase as a tracking gene under the CMV promoter. Co-transfection of each vector was performed with both miR-668-5p mimic and a negative control mimic (miC) at a concentration of 50 nM. After 48h, firefly and renilla luciferase activities were quantified in cell lysates using the DLR (Dual Luciferase Reporter) Assay System (E1910 - Promega, Madison, WI, USA) with readings taken on a FLUOstar Galaxy (BMG Labtechnologies, Ortenberg, Germany). Renilla luciferase served as a normalization control (12).

### 2.6 Protein extraction and Western blot analysis

After treatment and incubation, cell lysates were collected in 1× RIPA buffer (Merck Millipore, Burlington, MA, USA) with protease/phosphatase inhibitor cocktail (PIC) (Medchem Express, Monmouth Junction, NJ, USA). Similarly, femur bones were cleaned, ground in liquid nitrogen, and lysed in 1× RIPA buffer (Sigma, St. Louis, MO, USA) with PIC. Lysates were centrifuged (12,000 rpm, 20 min, 4 °C), and supernatants were collected for protein quantification using the Pierce™ BCA Protein Assay Kit (Thermo Scientific, Waltham, MA, USA). Protein samples were mixed with 4× loading dye, heat-denatured, and 25–30 µg was resolved via SDS-PAGE (Bio-Rad, Hercules, CA, USA) under reducing conditions. Proteins were transferred onto PVDF membranes (Merck Millipore, Burlington, MA, USA) using wet transfer, blocked with 5% BSA (Sigma, St. Louis, MO, USA) for 90 min, and incubated overnight at 4 °C with primary antibodies (**Supplementary Table S2**). After three TBST washes, membranes were incubated with HRP-conjugated secondary antibodies for 2h at room temperature, followed by additional washes. Signals were detected using a chemiluminescence system (Bio-Rad ChemiDoc XRS+ , Hercules, CA, USA), and band quantification was performed with ImageJ software.

### 2.7 Senescence-associated β-galactosidase staining

SA-β-gal staining was performed on miR-transfected MCOs treated with Doxo using the Senescence β-Galactosidase Staining Kit (9860 - Cell Signaling Technology CST, Danver, MA, USA) according to the manufacturer’s instructions. Senescent cells were visualized as blue stained under light microscopy. For quantification, cells were counted in three random fields per well to determine the percentage of SA-β-gal–positive cells.

### 2.8 Measurement of cellular ROS

To evaluate the Doxo-induced oxidative stress at the cellular level, the fluorescent probe DCFDA (2’-7’-dichlorodihydrofluorescein diacetate) dye (Sigma, St. Louis, MO, USA) was used for imaging (10×) (EVOS -Thermo Scientific, Waltham, MA, USA) and flow cytometry analysis (BD Biosciences FACSCalibur - San Diego ,CA,USA) using previously published standard protocols (29).

### 2.9 Experimental animals and model

The animal study followed ethical guidelines approved by the Institutional Animal Ethics Committee (IAEC) (Approval Reference No. IAEC/2022/61/Renew-1/Sr. No. 9 Dated: 21.09.2023) at CSIR-CDRI, Lucknow. All animal experiments were conducted in compliance with the guidelines of the Committee for the Control and Supervision of Experiments on Animals (CPCSEA), Government of India. Female Balb/c mice (22 ± 3 g, 12–16 weeks) from the National Laboratory Animal Centre were housed under controlled conditions with free access to food and water. To induce an accelerated aging phenotype, mice received intraperitoneal Doxo injections at either 2.5 mg/kg or 5 mg/kg on days 0 and 10, constituting two separate dose groups to determine the optimal dose of Doxo to induce cellular senescence *in vivo* (30–32). The control group received normal saline for 15 days. Based on pilot results, 5 mg/kg was finalized following significant changes observed in bone microarchitectural parameters and aging indices. For the miRNA study, mice were randomly divided into four different groups (*n* = 7): Vehicle (control animals administered only with vehicular solvent, i.e., normal saline), Doxo+miC, Doxo+miR-668-5p, and Doxo+anti-miR-668-5p and miRNA treatment began on day 15 post-Doxo. The animals further received subcutaneous injections of miC, miR-668-5p, and anti-miR-668-5p (Qiagen, Hilden, Germany), respectively, twice a week for three weeks. Each mouse was administered 100 µl of an oligonucleotide mixture containing 5 µg of oligonucleotide with an N/P ratio of 8. This corresponded to 0.16 µl of *in vivo*jetPEI (201-10G - Polyplus, Illkirch, France) per µg of oligonucleotide. In the fourth week, following echo MRI analysis, the mice were euthanized by gradual-fill controlled CO_2_ inhalation in compliance with the American Veterinary Medical Association (AVMA) and CPCSEA guidelines. The femur bones, vertebrae, and serum samples were collected for further experimental analysis. The body weight of the animals was monitored and recorded throughout dosing.

### 2.10 Micro-CT analysis

Femur bones and L5 vertebrae were analyzed using a high-resolution micro-computed tomography (µCT) scanner (Skyscan 1076 - Aartselaar, Antwerp, Belgium) following a standard published protocol (33). In brief, trabecular areas of bones were scanned at 70 kV and 100 mA with 18 mm resolution. From 100 projections spanning 180°, image slices were generated and reconstructed in Sky Scan NRecon software with a modified Feldkamp algorithm. Scans were then analyzed using CTAn analysis software. The µCT data facilitated the generation of three-dimensional (3D) reconstructions of the trabecular network. Trabecular microarchitecture parameters (BV/TV, Tb.N, Tb.Th, Tb.Sp, BS/TV, SMI, Tb.pf) were assessed, and femoral trabecular BMD was measured using calibrated hydroxyapatite rods.

### 2.11 Bone strength testing

Bone strength was assessed using a three-point bending method following a standardized protocol (34) in a bone strength testing apparatus (TK252C - Muromachi Kikai, Japan). Mechanical strength parameters like power, energy, and stiffness were subsequently calculated based on load-displacement curves generated during testing.

### 2.12 Analysis of serum biochemical markers

Prior to autopsy, animals were fasted for 5h–6h before blood collection was performed. Serum levels of key bone turnover biomarkers, including amino-terminal propeptide of type I procollagen (P1NP) and C-terminal telopeptide of type I collagen (CTx), were quantified using enzyme-linked immunosorbent assay (ELISA) kits (E-EL-M0233, E-EL-M3023 - Elabscience, Wuhan, China). Total Superoxide Dismutase (T-SOD) Activity Assay Kit (E-BC-K020-M - Elabscience, Wuhan, China) was employed to check the total SOD activity in serum samples. The assays were carried out following the manufacturer’s protocols.

### 2.13 Bone dynamic histomorphometry

Bone dynamic histomorphometry was assessed via calcein double labeling. Mice received intraperitoneal calcein (20mg/kg; Sigma, St. Louis, MO, USA) twice, first before miRNA dosing and second 48h before autopsy. In brief, after autopsy, femur bones were harvested, cleaned, embedded in acrylic material, sectioned at 50 μm using an Isomet bone cutter, and further examined under a fluorescence microscope (EVOS -Thermo Scientific, Waltham, MA, USA). Measurements were carried out using ImageJ software, and standard indices of mineralizing surface (MS/BS), mineral apposition rate (MAR), and bone formation rate (BFR) were derived according to established protocol (29).

### 2.14 Trap staining

For the detection of osteoclasts, tartrate-resistant acid phosphatase (TRAP) staining was performed on decalcified paraffin-embedded femoral sections. Briefly, mice femur sections (5 µm) were deparaffinized in xylene and rehydrated through graded ethanol to distilled water. Following previously published protocol (35), the dewaxed tissue sections were first preincubated with a Naphthol AS-BI Phosphate Substrate solution (Sigma, St. Louis, MO, USA), after which they were treated with a mixture of Sodium Nitrite (Sigma, St. Louis, MO, USA) and Pararosaniline Dye (Sigma, St. Louis, MO, USA). Sections were then rinsed in distilled water, counterstained with 0.02% fast green, dehydrated through graded alcohols, cleared in xylene, and lastly mounted for microscopic examination. TRAP-positive cells were identified as osteoclasts and quantified using ImageJ software.

### 2.15 Statistical analysis

Data are presented as mean ± S.E.M., with *p < 0.05, **p < 0.01, and ***p < 0.001 considered significant. One-way ANOVA followed by the Newman–Keuls test of significance for multiple group comparisons, and Student’s t-test for two-group comparisons was carried out using GraphPad Prism 5.0. Experiments were repeated at least three times, presenting representative data.

## 3 Results

### 3.1 miR-668-5p positively regulates osteoblast function

**Figure 1:**
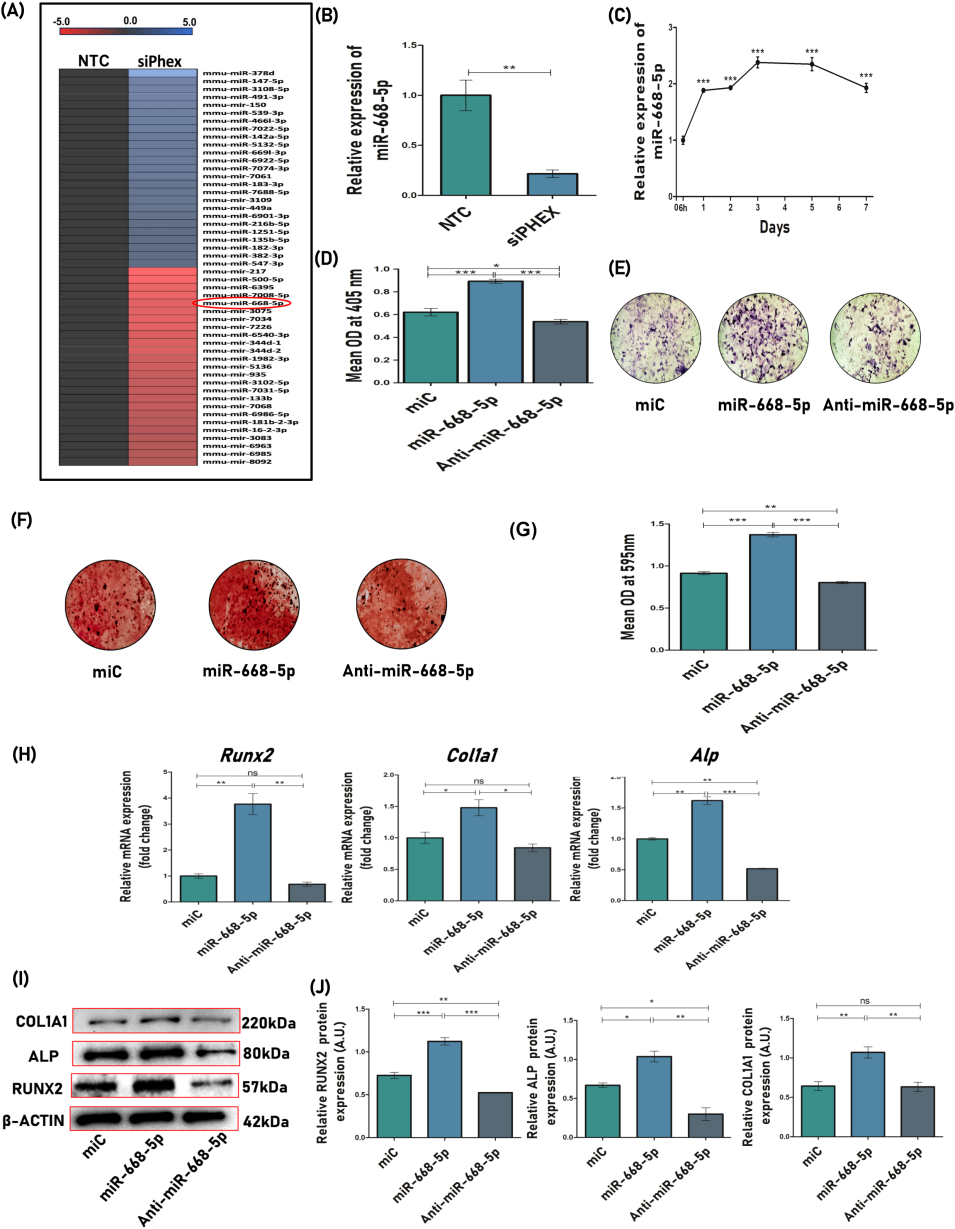
miR-668-5p positively regulates osteoblast function. **(A)** miRNA expression profile displaying representative miRNAs with significant upregulation and downregulation in Phex-silenced osteoblasts (NTC-non-targeted control siRNA; siPhex-Phex siRNA). Expression levels are shown relative to the median, with blue indicating high expression and red representing low expression. **(B)** miR-668-5p expression in siPhex transfected osteoblast cells after 48h of transfection. **(C)** Temporal changes in miR-668-5p expression during osteoblast differentiation. **(D)** ALP assay in MCO(s) treated with negative control (miC), miR-668-5p mimic, or anti–miR-668-5p in osteoblast differentiation medium after 48h of transfection. **(E)** Representative images of ALP staining (4×). **(F)** Representative images of ARS staining (4×) and **(G)** quantification of ARS by CPC method (OD = 595nm). **(H)** qPCR analysis of osteogenic markers (Runx2, Col1a1 & Alp) normalized to Gapdh. **(I)** Western blots of osteogenic markers (Runx2, Col1a1 & Alp) normalized to β-actin, after 48h of transfection and **(J)** densitometric analysis of Western blots using ImageJ software. Data are expressed as mean ± SEM (*n* = 3); *p < 0.05, **p < 0.01, ***p < 0.001, ns (non-significant) compared between groups.

Previous miRNA profiling data in Phex-silenced osteoblast cells identified several miRNA candidates to be differentially regulated (12) (**Figure 1A**). The miRNA profiling data was firstly validated with quantitative real-time PCR (qPCR), which showed approximately fivefold downregulated expression of miR-668-5p in siPhex-treated osteoblast cells (**Figure 1B**). Furthermore, the expression of miR-668-5p was found to be increased in mouse calvarial osteoblasts (MCOs) during osteoblast differentiation (**Figure 1C**).

After confirming the profiling data, the effect of miR-668-5p on osteoblast differentiation and mineralization was evaluated. MCOs were transfected with 30 nM of miC (negative control), 50 nM of miR-668-5p (mimic), and 50 nM of anti-miR-668-5p (inhibitor) and were subjected to ALP (alkaline phosphatase) measurement after 48h. ALP activity, an initial and key marker of osteoblast differentiation (36), was found to be most upregulated in miR-668-5p transfected cells. Conversely, the ALP activity was significantly reduced in cells transfected with anti-miR-668-5p (**Figure 1D**). Similar results were seen with the ALP staining in a 7-day culture (**Figure 1E**). Following the ALP assessment, the effect of miR-668-5p was checked on the mineralization potential of MCOs. The overexpression of miR-668-5p significantly increased the mineral nodule formation in osteoblasts in the 18-day culture. Moreover, transfection with anti–miR-668-5p significantly decreased mineral nodule formation (**Figures 1F, G**). These results indicate miR-668-5p as a positive regulator of osteoblast differentiation and mineralization.

The next step was to investigate the effect of miR-668-5p on osteogenic markers. Various osteogenic markers like Runx2 (runt-related transcription factor 2), Col1a1 (collagen type I alpha 1 chain), and Alp, and so forth, are actively involved in the process of osteoblast formation, differentiation, and bone formation (37). The effect of miR-668-5p on these osteogenic factors was determined. The mRNA expression of osteogenic genes Runx2, Alp, and Col1a1 was found to be significantly upregulated in miR-668-5p–transfected cells (**Figure 1H**). A similar effect was observed at the translational level, where miR-668-5p transfection increased the expression of these markers, which was reversed by anti–miR-668-5p transfection in osteoblast cells (**Figures 1I, J**). This data highlights the potential of miR-668-5p in regulating the expression of osteogenic markers at both transcriptional and translational levels.

### 3.2 miR-668-5p directly targets E3 ubiquitin ligase Znrf3 and upregulates the Wnt/β-catenin signaling pathway in osteoblasts

**Figure 2:**
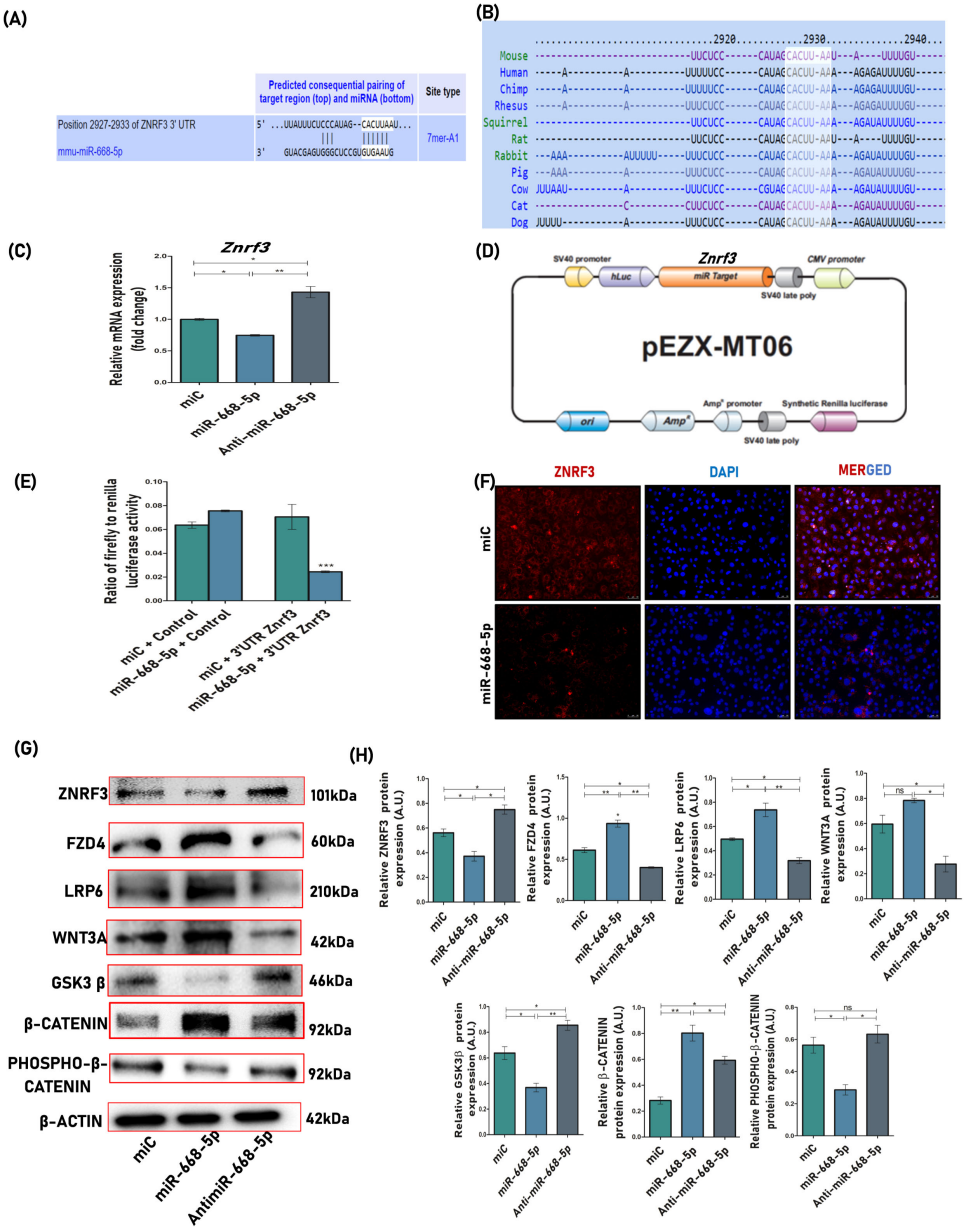
miR-668-5p directly targets E3 ubiquitin ligase Znrf3 and upregulates the Wnt/β-catenin signaling pathway in osteoblasts. **(A)** Target screening via computational analysis showing the complementary sequences of miR-668-5p to the 3′UTR of Znrf3 **(B)** The target sequence is conserved across mammalian species. **(C)** qPCR analysis of Znrf3 normalized to Gapdh after 48h of transfection. **(D)** Schematic representation of the reporter plasmid pEZX-MT06 used to examine the effect of the Znrf3 3′UTR on luciferase activity. **(E)** Target validation by DLR (Dual Luciferase Reporter) assay. Cells were cotransfected with either 3′UTR Znrf3 clone or control clone and miR-668-5p or miC. Firefly and renilla luciferases were quantified in transfected cell lysates. **(F)** Representative images (20×) of Immunofluorescence staining showing Znrf3 expression (red) in miC and miR-668-5p transfected cells, with nuclear marker DAPI (blue). **(G)** Western blots of proteins involved in Wnt/β-catenin signaling pathway (Znrf3, Fzd4, Lrp6, Wnt3a, Gsk3β, β-catenin & Phospho-β-catenin) normalized to β-actin and **(H)** densitometric analysis of Western blots. Data are expressed as mean ± SEM (*n* = 3); *p < 0.05, **p < 0.01, ***p < 0.001, ns (non-significant) compared between groups.

Our subsequent analysis focused on uncovering the molecular mechanism by which miR-668-5p regulates osteoblast function. To identify potential target genes for miR-668-5p, target prediction tools such as TargetScan and miRDB were employed. Among the numerous genes predicted as potential targets by these databases, our focus was specifically directed toward Znrf3, an E3 ubiquitin ligase that acts as a negative regulator of the Wnt signaling pathway (38). Moreover, it contains one 7-mer A1 sequence similarity site associated with miR-668-5p in the seed sequence, which is also conserved across different mammalian species (**Figures 2A, B**). Therefore, we aimed to confirm whether Znrf3 is a potential target of miR-668-5p. MCOs were first transfected with miC, miR-668-5p, and anti–miR-668-5p, and the transcript levels of Znrf3 were checked. The results showed a significant reduction in Znrf3 mRNA expression in miR-668-5p transfected cells, while this effect was reversed in anti-miR-668-5p transfected cells (**Figure 2C**). Further, to confirm the target specificity, a DLR assay was performed. First, two luciferase reporter constructs were synthesized, one containing the 3′-UTR of Znrf3 (**Figure 2D**) and the other lacking the 3′-UTR (control/empty clone). These constructs were co-transfected into osteoblast cells with either miR-668-5p or miC, and enzymatic activity of both Firefly (primary reporter) and Renilla (internal standard) luciferases was measured. Overexpression of miR-668-5p significantly suppressed the luciferase activity of the reporter gene in Znrf3-3′UTR transfected cells, whereas no change in activity was observed when miR-668-5p was co-transfected with the control clone (lacking 3’UTR), thereby confirming the target specificity (**Figure 2E**). Immunofluorescence staining also showed minimal expression of Znrf3 with reduced red fluorescence intensity in miR-668-5p-treated cells as compared to miC, confirming Znrf3 as the direct functional target of miR-668-5p (**Figure 2F**).

Since Znrf3 was found to be a confirmed target for miR-668-5p, it was interesting to check its downstream signaling mechanism. Znrf3, a Wnt signaling antagonist, functions to ubiquitinate and promote subsequent degradation of WNT receptor components Frizzled (FZD) and Low-Density Lipoprotein Receptor-Related Protein 6 (LRP6), thereby forming a core negative feedback circuit in the Wnt/β-catenin signaling pathway (39). Protein lysates were collected from miRNA-transfected cells and analyzed using antibodies targeting the Wnt/β-catenin signaling pathway. Initial assessment revealed a significant downregulation of Znrf3 protein levels in miR-668-5p transfected cells, which supported our previous findings. Subsequent Western blot analysis demonstrated that miR-668-5p mimic transfection led to increased protein levels of Fzd4, Lrp6, Wnt3a, and β-catenin compared to the miC group. Conversely, phospho-β-catenin and Gsk3β protein levels were downregulated in miR-668-5p mimic-transfected cells. In contrast, treatment with anti–miR-668-5p induced the opposite effects on the expression of these proteins, highlighting the role of miR-668-5p in enhancing the Znrf3-mediated Wnt/β-catenin signaling pathway (**Figures 2G, H**).

### 3.3 miR-668-5p overexpression mitigates doxorubicin-induced osteoblast senescence

The study simultaneously investigated whether overexpressing or inhibiting miR-668-5p had any regulatory effects on cellular senescence and the aging process of MCOs. To do so, the widely used chemotherapeutic agent doxorubicin (Doxo) was utilized, which is known to induce cellular senescence in various cell types (22). An effective sublethal dose of Doxo, that is, 25 nM, was determined through ALP, MTT, Western blotting, and SA-β-gal assay (**Supplementary Figure S1**), which was sufficient to induce cellular senescence and inhibit osteoblast differentiation in MCOs.

**Figure 3:**
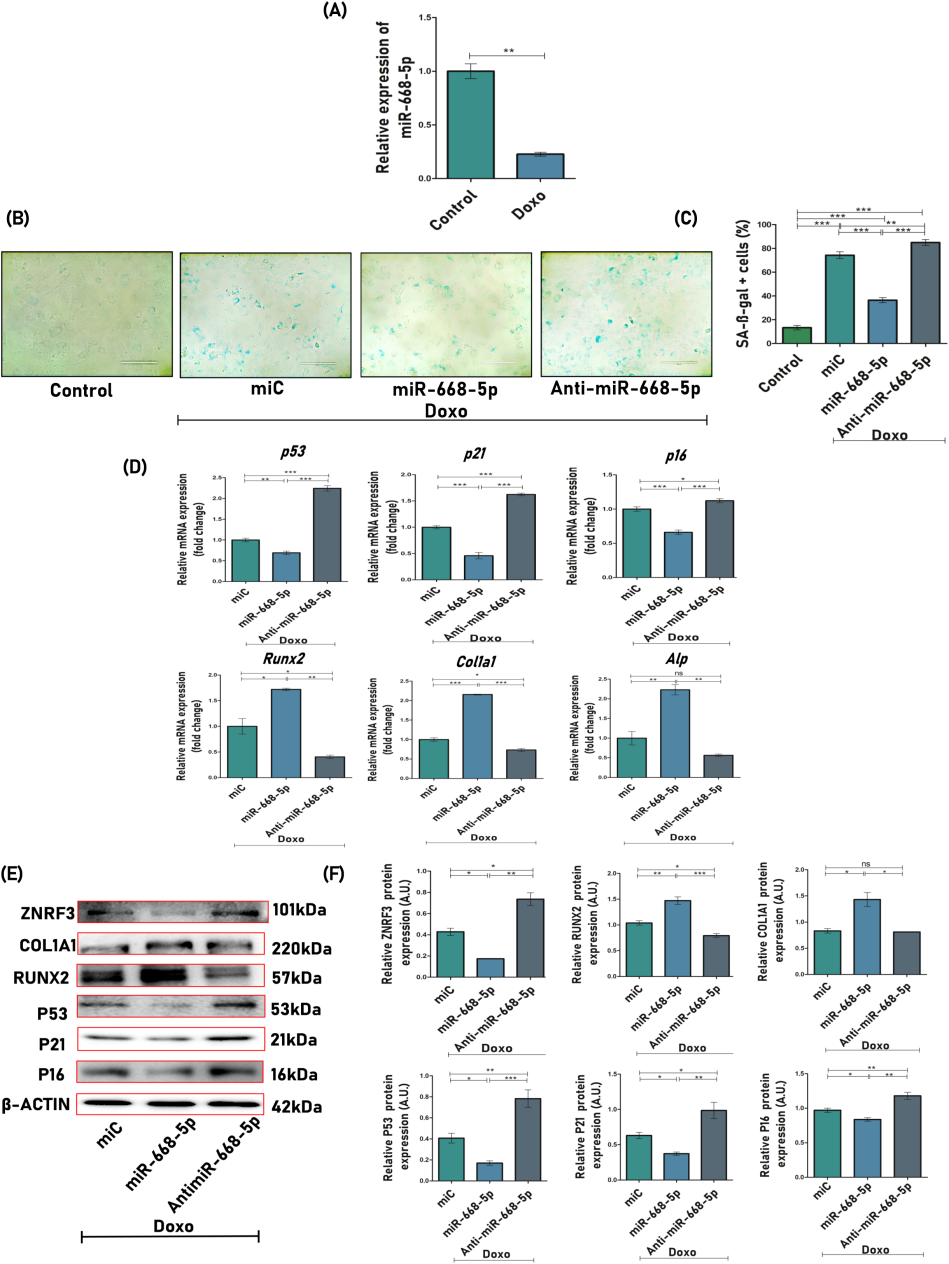
miR-668-5p overexpression mitigates doxorubicin-induced osteoblast senescence. **(A)** miR-668-5p expression in Doxo (Doxorubicin) treated cells. **(B)** Representative images (20×) of SA-β-gal (Senescence-associated β-galactosidase) staining in miR-transfected MCOs in the presence of Doxo, along with untreated control and **(C)** SA-β-gal-positive cells (%) quantification. **(D)** qPCR analysis of senescence-associated (p53, p21 & p16) and osteogenic genes (Runx2, Col1a1, Alp) in miR-transfected cells in the presence of Doxo (Gapdh, internal control). **(E)** Western blots of Znrf3, osteogenic (Runx2 & Col1a1), and senescence-associated proteins (p53, p21, & p16) (β-actin, internal control) and **(F)** densitometric analysis of Western blots. Data are expressed as mean ± SEM (*n* = 3); *p < 0.05, **p < 0.01, ***p < 0.001, ns (non-significant) compared between groups.

First, qPCR analysis revealed that endogenous levels of miR-668-5p were significantly downregulated in Doxo-treated MCOs (**Figure 3A**). To analyze whether miR-668-5p was playing a role in Doxo-induced cellular senescence, SA-β-gal (senescence-associated β-galactosidase) staining was employed to study aging-related physiology. Compared to miC+Doxo (74%), the cells in the miR-668-5p (mimic)+Doxo-treated group exhibited decreased levels of SA-β-gal–positive cells (36%), while their proportion was highest in the anti-miR-668-5p+Doxo–treated group (85%) (**Figures 3B, C**). Further, the mRNA expression of senescence-associated cell cycle markers like p53, p21, and p16 was significantly downregulated in miR-668-5p–transfected cells as compared to miC (control). Additionally, the mRNA expression of osteogenic markers like Runx2, Col1a1, and Alp was also checked to understand the effect of Doxo on osteoblast function. Results show that the overexpression of miR-668-5p alleviated the inhibition of osteoblast differentiation induced by Doxo. These effects were reversed in cells transfected with anti–miR-668-5p (**Figure 3D**). This counteracting effect of miR-668-5p against Doxo was also checked at the translational level. The protein levels of senescence (p53, p21, and p16) and osteogenic markers (Runx2 and Col1a1) were checked via Western blotting. The results were found to be consistent with qPCR (**Figures 3E, F**).

### 3.4 miR-668-5p overexpression counters doxorubicin-induced ROS production and mitochondrial dysfunction

**Figure 4:**
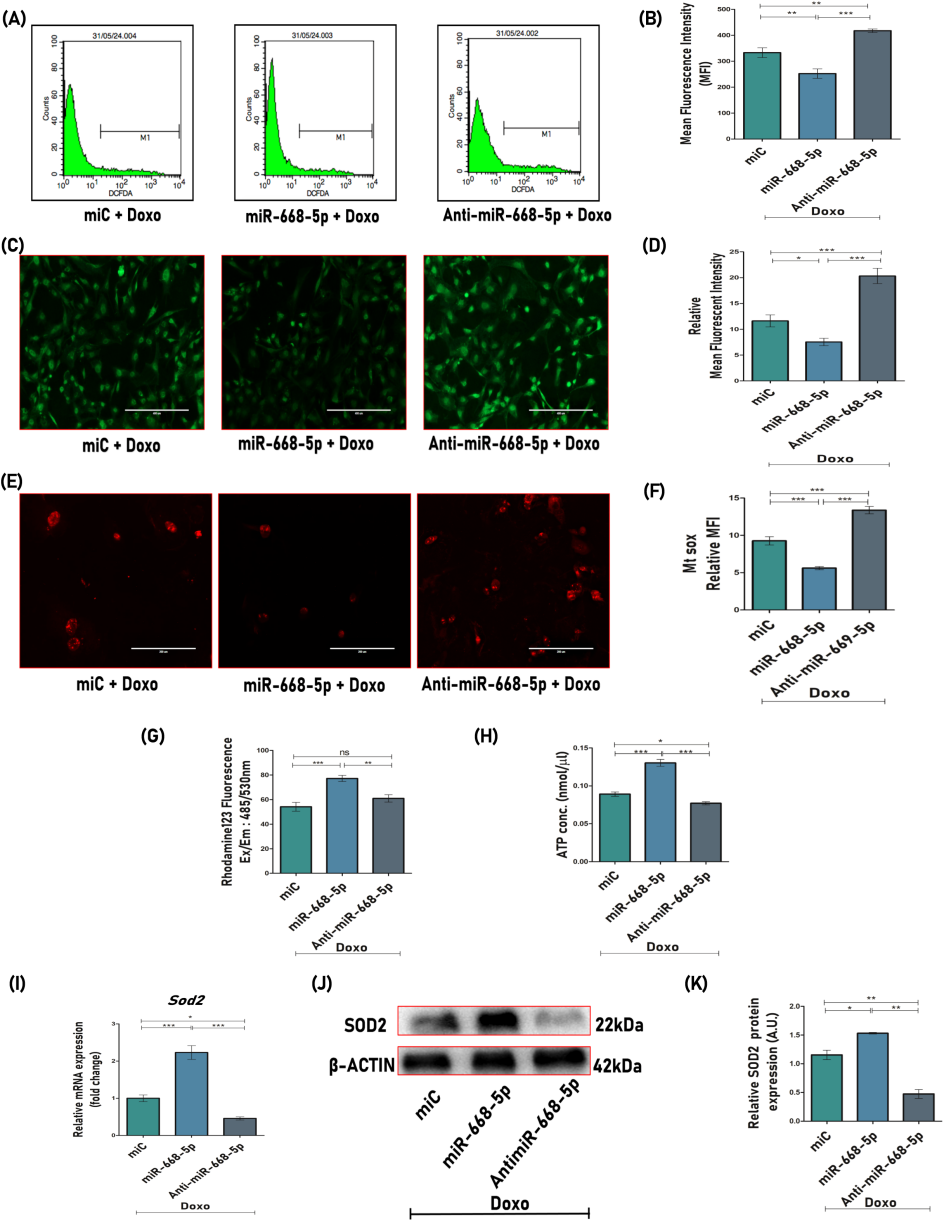
miR-668-5p overexpression counters doxorubicin-induced ROS production and mitochondrial dysfunction. **(A)** Cellular ROS analysis and **(B)** quantification using DCFDA flow cytometry. **(C)** Representative images (10×) of DCFDA staining and **(D)** mean fluorescent intensity (MFI) quantification. **(E)** Representative MitoSOX Red images (20×) and **(F)** MFI quantification. **(G)** MMP assessment using Rhodamine 123 fluorescent dye and **(H)** ATP production assessment using ATP assay kit. **(I)** qPCR analysis (Gapdh, internal control) and **(J)** Western Blot (β-actin, internal control) of antioxidant enzyme Sod2 in miR transfected cells. **(K)** Densitometric analysis of Western blot. Data are expressed as mean ± SEM (*n* = 3); *p < 0.05, **p < 0.01, ***p < 0.001, ns (non-significant) compared between groups.

Doxorubicin has long been recognized for its ability to induce intracellular reactive oxygen species (ROS) generation, which accumulates over time, thereby triggering oxidative stress and subsequent cellular damage (40). The effect of miR-668-5p on the mitigation of cellular oxidative stress induced by Doxo was investigated. It is evident from the data that both miC+Doxo and anti-miR-668-5p+Doxo-treated cells exhibited elevated ROS production at cellular levels as examined by DCFDA flow cytometry (**Figures 4A, B**) and staining (**Figures 4C, D**), while this overproduction of ROS was significantly decreased in cells treated with miR-668-5p in Doxo conditions.

To further investigate mitochondrial ROS levels in miR-transfected cells in the presence of Doxo, live-cell imaging was performed using MitoSOX Red, a mitochondrial superoxide-specific dye. MitoSOX Red fluorescence was found to be significantly reduced in miR-668-5p-transfected cells in the presence of Doxo, suggesting lower mitochondrial superoxide levels as compared to miC (**Figures 4E, F**). In contrast, anti-miR treatment exhibited the opposite effect. Elevated ROS levels can disrupt mitochondrial functions, including mitochondrial membrane potential (MMP) and ATP synthesis. This was assessed using Rhodamine 123 staining and ATP assay, where Doxo treatment induced mitochondrial dysfunction by reducing MMP and ATP levels. However, miR-668-5p overexpression effectively restored MMP and ATP levels as compared to the miC and anti–miR-668-5p groups (**Figures 4G, H**). Notably, miR-668-5p+Doxo–treated cells showed significant upregulation of the mitochondrial antioxidant enzyme Sod2 (superoxide dismutase 2) at both transcriptional and translational levels (**Figures 4I–K**), highlighting its potential role in maintaining redox homeostasis of the cells.

In line, we checked how miR-668-5p was engaged in cell survival under the influence of Doxo. Treated MCOs were double stained with Annexin V-FITC/PI and analyzed by flow cytometry. The results showed that in Doxo conditions, miR-668-5p overexpression protected cells from entering early apoptosis as compared to anti–miR-668-5p treatment, which resulted in a higher proportion of apoptotic cells (**Supplementary Figure S2**). The relative mRNA and protein levels of apoptosis regulators Bax and Bcl2 were also checked. In anti–miR-668-5p transfected cells, we observed increased Bax and decreased Bcl2 levels compared to both miC and mimic transfected cells (**Supplementary Figure S2**), underscoring the anti-apoptotic role of miR-668-5p in regulating osteoblast survival.

### 3.5 miR-668-5p overexpression ameliorates trabecular microarchitecture and bone quality in doxorubicin-administered mice

Our *in-vitro* findings demonstrated that miR-668-5p exhibits osteoprotective and anti-senescent properties against Doxo in primary osteoblast cells. These results prompted us to extend our investigation into an *in-vivo* model of aging-induced bone loss. A mouse model of Doxo-induced accelerated aging was developed for this purpose. Based on microCT analysis and Western blot expression of osteogenic and senescence markers, the 5 mg/kg dose of Doxo was identified as optimal for inducing bone loss and aging-associated skeletal changes (**Supplementary Figure S3**).

**Figure 5:**
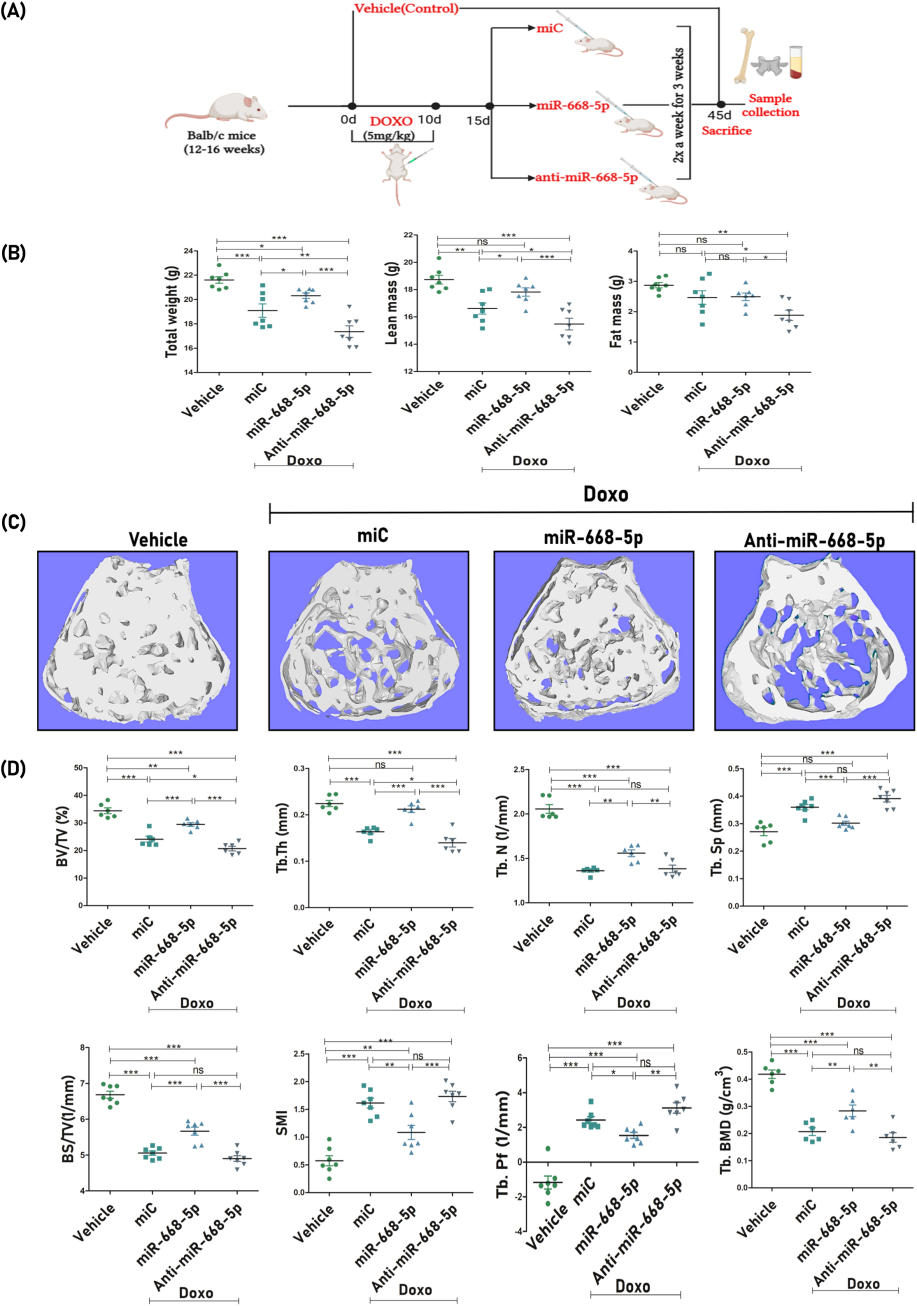
miR-668-5p overexpression ameliorates femoral trabecular microarchitecture in doxorubicin-administered mice. **(A)** *In-vivo* study design. **(B)** Echo MRI analysis of body composition showing Total body weight, Lean Mass, and Fat Mass (g) in different animal groups. **(C)** Representative 3D micro-CT images of femur trabecular network. **(D)** Quantification of femoral trabecular microarchitecture parameters (BV/TV%, Tb.Th., Tb.N., Tb.Sp., BS/TV, SMI & Tb.pf.) and bone mineral density (Tb. BMD). Data are expressed as mean ± SEM (*n* = 6); *p < 0.05, **p < 0.01, ***p < 0.001, ns (non-significant) compared between groups.

For the miRNA study, Doxo-treated mice were subsequently administered with miC (negative control), miR-668-5p (mimic), and anti–miR-668-5p (inhibitor) for 3 weeks. The *in-vivo* study design is represented in **Figure 5A**. A day before the autopsy, an echo MRI for body composition was carried out, which revealed that Doxo administration caused a significant reduction in total body weight and lean mass compared to the vehicle-treated group. However, post-treatment, the miR-668-5p group exhibited a moderate restoration of body weight and lean mass as compared to both the miC and anti-miR groups. Changes in fat mass were not statistically significant, although animals treated with anti–miR-668-5p exhibited the lowest body weight, lean mass, and fat mass composition (**Figure 5B**).

After autopsy, bone samples were collected, and subsequent analysis focused on trabecular bone parameters at the femur metaphysis and L5 vertebrae. Representative 3D micro-CT images (**Figure 5C**) of femoral trabeculae revealed comparative levels of trabecular bone loss across the Doxo-treated miR groups. Quantitative micro-CT analysis was conducted to assess key trabecular bone parameters. Results showed that miR-668-5p administration significantly mitigated Doxo-induced bone loss. This was evidenced by improvements in bone volume per tissue volume (BV/TV%), trabecular number (Tb.N.), and trabecular thickness (Tb.Th.) alongside reductions in trabecular separation (Tb.Sp.) as compared to the miC group. Additionally, trabecular indices, including bone surface density (BS/TV), structure model index (SMI), and trabecular bone pattern factor (TB.pf.), were assessed. In the miR-668-5p group, BS/TV was increased, while SMI and Tb.Pf. were reduced compared to the miC and anti-miR groups. Furthermore, femoral trabecular bone mineral density (BMD) was significantly restored in the mimic-treated animals. In contrast, the anti-miR-668-5p treatment exacerbated trabecular bone loss, reversing the protective effects of miR-668-5p (**Figure 5D**).

**Figure 6:**
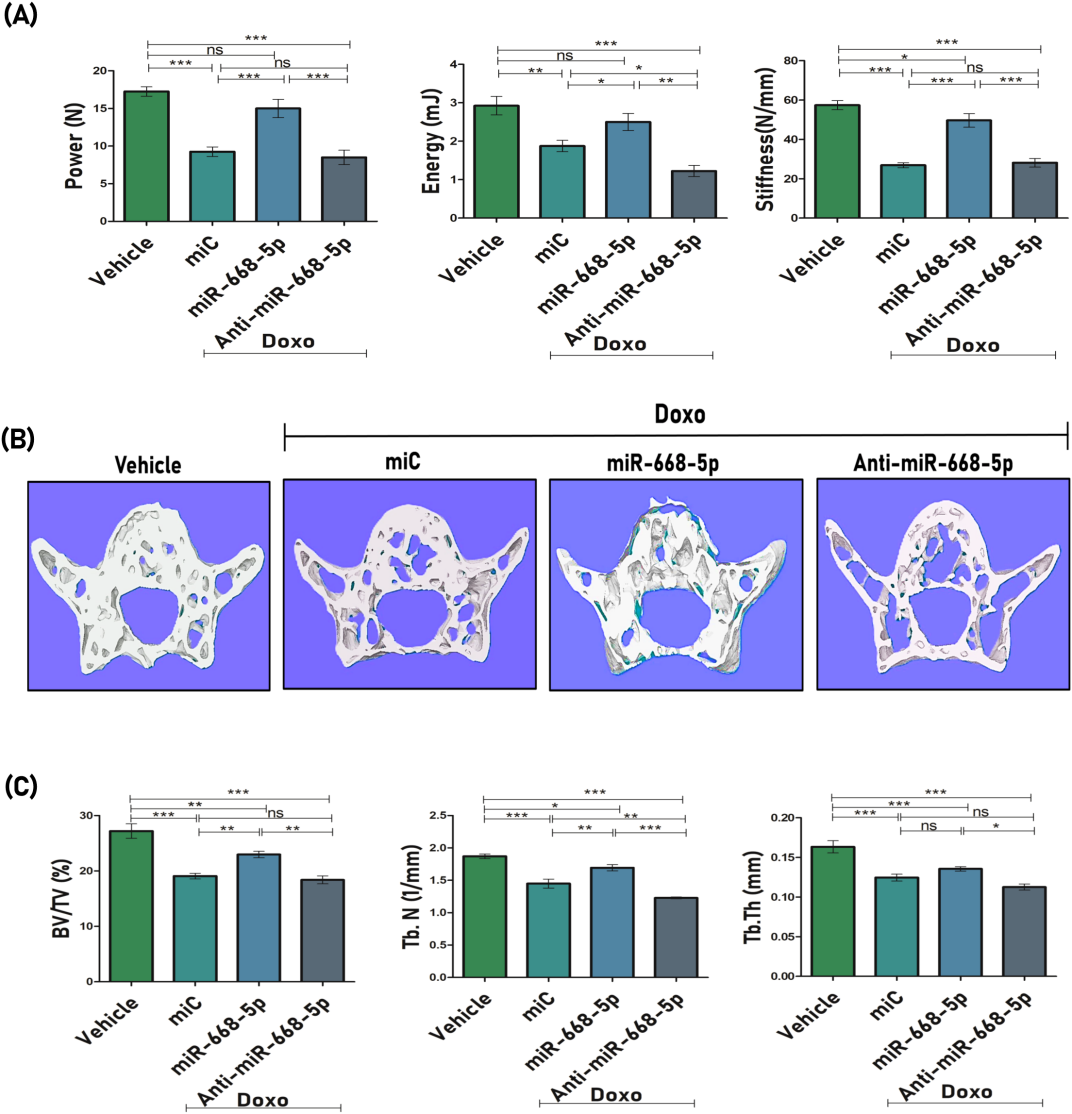
miR-668-5p overexpression improves bone strength and vertebral bone microarchitecture in doxorubicin-administered mice **(A)** Biomechanical strength parameters analysis of mice femur showing Power (N), Energy (mJ) & Stiffness (N/mm) using three-point bending test. **(B)** Representative 3D micro-CT images of Lumbar 5 (L5) trabecular network and **(C)** quantification of L5 trabecular microarchitecture parameters (BV/TV%, Tb.N., & Tb.Th.). Data are expressed as mean ± SEM (*n* = 6); *p < 0.05, **p < 0.01, ***p < 0.001, ns (non-significant) compared between groups.

The impact of miR-668-5p on the biomechanical strength of the femoral bone was also evaluated. Doxo-treated animals showed compromised bone strength, as determined by biomechanical parameters such as power (N), energy (mJ), and stiffness (N/mm) using a three-point bending test. miR-668-5p–treated bone samples displayed significantly improved bone strength across these parameters compared to both the miC and anti–miR-668-5p groups (**Figure 6A**). Later, we also evaluated bone loss at the primary load-bearing L5 vertebrae, where miR-668-5p ameliorated Doxo-induced bone loss by enhancing BV/TV and Tb.N parameters. Tb.Th was also enhanced, but the observed change was non-significant as compared to the miC group. (**Figures 6B, C**).

### 3.6 Overexpression of miR-668-5p enhances bone mineralization and regeneration and modulates biochemical, osteogenic, and senescence markers in doxorubicin-administered mice

**Figure 7:**
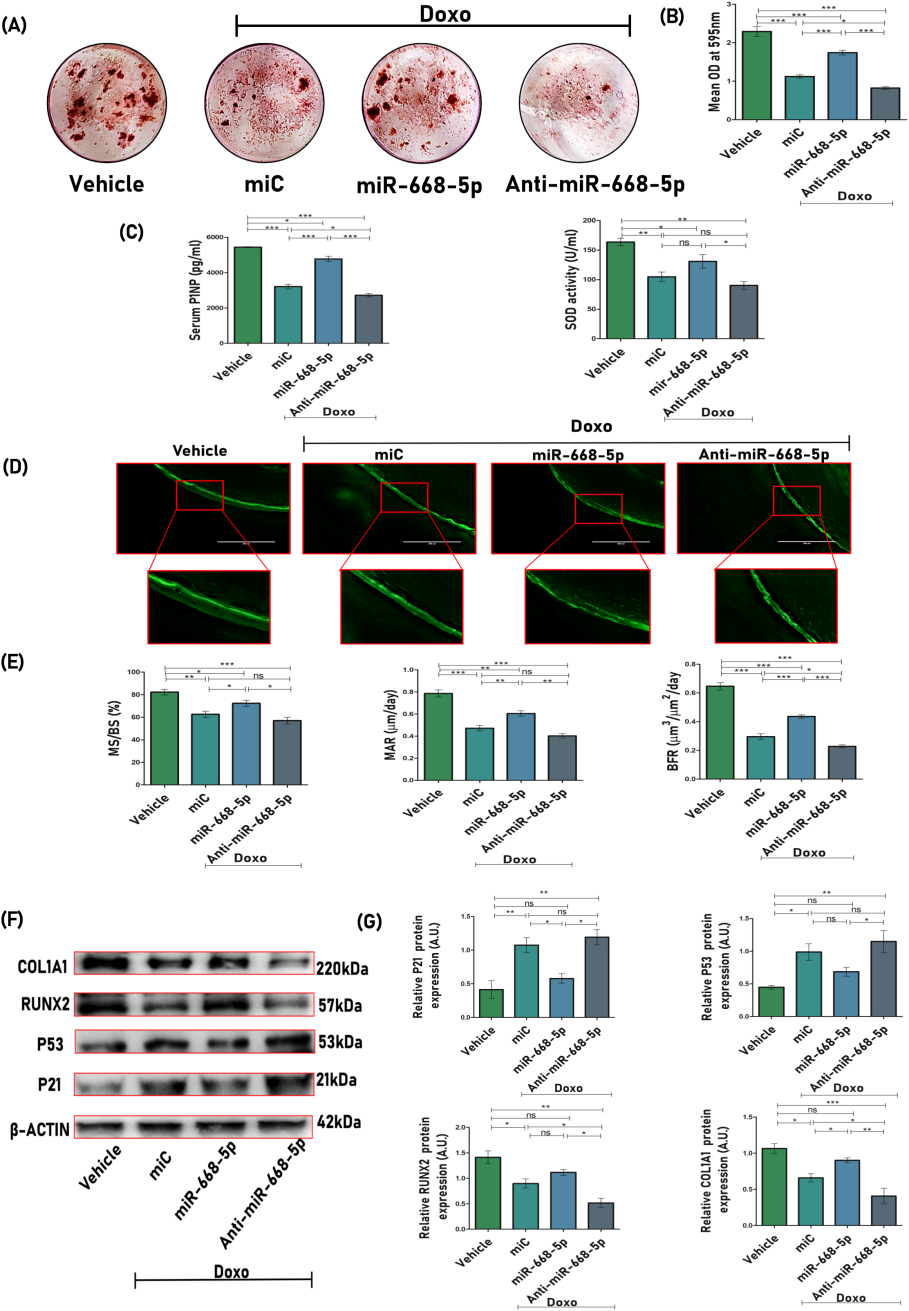
Overexpression of miR-668-5p enhances bone mineralization, regeneration, and modulates biochemical, osteogenic, and senescence markers in doxorubicin-administered mice. **(A)** Representative ARS staining images of BMSCs displaying *ex-vivo* mineralization among different animal groups and **(B)** quantification of ARS by CPC method (OD = 595nm). **(C)** Serum P1NP levels and SOD activity in mice serum samples. **(D)** Representative images (20×) of the femur diaphysis (transverse sections) showing calcein double labeling. **(E)** Bone histomorphometric parameters assessed from calcein labeling experiments, including %MS/BS (mineralizing surface), MAR (mineral apposition rate), and BFR (bone formation rate). **(F)** Western blots of osteogenic (Runx2 & Col1a1) and senescence-associated markers (p53 & p21) in femur bone samples (β-actin, internal control) and **(G)** densitometric analysis of Western blots. Data are expressed as mean ± SEM (*n* = 6); *p < 0.05, **p < 0.01, ***p < 0.001, ns (non-significant) compared between groups.

Next, we checked the efficacy of miRNA treatment on osteogenic differentiation and mineralization. *Ex-vivo* mineralization assay was performed, and results showed significant mineral nodule formation in miR-668-5p–treated bone marrow stromal cells (BMSCs) as compared to the miC group, while anti–miR-668-5p treatment significantly reduced nodule formation (**Figures 7A, B**). The impact of miR-668-5p on biochemical markers was also investigated. Serum levels of P1NP (procollagen type 1 N-terminal propeptide), a marker of bone formation (41), were measured using sandwich ELISA. Serum samples of miR-668-5p–administered animals demonstrated increased P1NP levels compared to the miC group, whereas these effects were reversed in the anti-miR-668-5p–treated group. Additionally, SOD (superoxide dismutase) activity was evaluated in the serum samples, and it was found to be relatively higher in the mimic-treated animal group compared to both the miC and anti-miR-treated groups, though the increase was not statistically significant compared to the miC group (**Figure 7C**). The efficacy of bone regeneration following Doxo administration was evaluated using dynamic histomorphometry of the femoral diaphysis across different treatment groups. Representative images revealed intense and continuous calcein labeling with closely opposed double labeling in the vehicle group, compared to diminished labeling in the Doxo-treated groups. Among these, miR-668-5p–administered bone samples displayed the least compromised calcein double labeling, indicating a protective effect (**Figure 7D**). Quantitative analysis showed that miR-668-5p treatment significantly improved %MS/BS (mineralizing surface), MAR (mineral apposition rate), and BFR (bone formation rate) compared to miC and anti-miR-668-5p–treated groups (**Figure 7E**). These results collectively indicate that miR-668-5p administration mitigates Doxo-induced bone damage by enhancing bone formation and mineralization while modulating serum markers of bone turnover and antioxidant activity.

To investigate the role of miR-668-5p in modulating the protein expression of osteogenic and senescence-associated markers, femoral bone sample lysates were subjected to Western blot analysis. Treatment with miR-668-5p resulted in reduced expression of the senescence markers p53 and p21 while significantly restoring the osteogenic markers Runx2 and Col1a1 compared to miC-treated samples. Conversely, these effects were reversed in the anti-miR-668-5p–treated group (**Figures 7F, G**). These results reflect the protective role of miR-668-5p in aging bone loss conditions.

### 3.7 Overexpression of miR-668-5p decreases expression of osteoclastogenic and adipogenic markers

**Figure 8:**
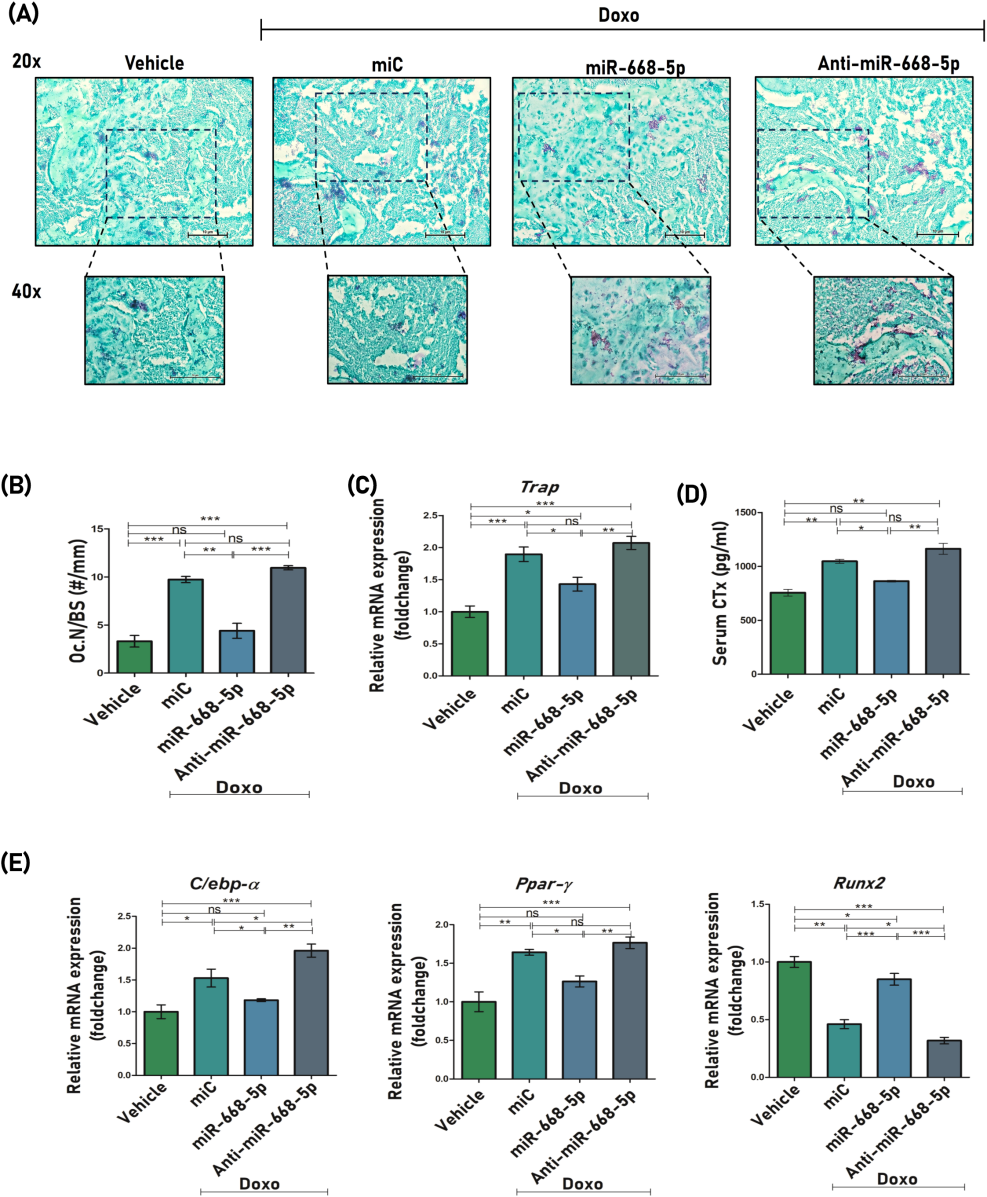
Overexpression of miR-668-5p decreases expression of osteoclastogenic and adipogenic markers. **(A)** Representative TRAP staining images of femur section (20× and 40×). **(B)** Quantification of osteoclast numbers per mm of bone surface, that is, Oc. N/BS (#/mm). **(C)** qPCR analysis of Trap mRNA levels in mice femur samples (Gapdh, internal control). **(D)** Serum CTx levels **(E)** qPCR analysis of C/EBPα, PPARγ and Runx2 in mice femur samples (Gapdh, internal control). Data are expressed as mean ± SEM (*n* = 3); *p < 0.05, **p < 0.01, ***p < 0.001, ns (non-significant) compared between groups.

To assess the anti-resorptive effects of miR-668-5p, TRAP (tartrate-resistant acid phosphatase) staining was conducted on femoral bone sections. Consistent with doxorubicin’s established role in promoting osteoclastogenesis and bone loss (42), Doxo+miC-treated samples exhibited a higher number of TRAP-positive osteoclasts per bone surface (Oc.N/BS). Interestingly, treatment with miR-668-5p mimic markedly reduced the number of TRAP-positive cells (**Figures 8A, B**). This histological observation was supported at the molecular level, with TRAP mRNA expression showing a similar trend across groups (**Figure 8C**). Additionally, a significant decrease in serum CTx (C-terminal telopeptide of type I collagen), a marker of bone resorption, was observed in serum samples of miR-668-5p–administered mice (**Figure 8D**). Together, these results indicate a potential attenuating effect of miR-668-5p on Doxo-induced osteoclast activity and bone resorption.

With advancing age, bone formation declines significantly, largely due to a shift in bone marrow stem cell differentiation from osteoblastogenesis toward increased adipogenesis (43). The effect of miR-668-5p was therefore also studied on the mRNA expression levels of key adipogenic regulators like C/EBP-α (CCAAT/enhancer-binding protein alpha) and PPARγ (peroxisome proliferator-activated receptor gamma), along with the osteogenic factor Runx2 in mice femur samples. The Doxo+miR-668-5p group samples showed slightly reduced expression levels of PPARγ and C/EBPα compared to the Doxo+miC and Doxo+Anti-miR-668-5p groups. Runx2 levels, on the other hand, were restored in the miR-668-5p group (**Figure 8E**). These findings suggest a potential role of miR-668-5p in the regulation of age-induced adipogenic activity.

### 3.8 Znrf3 overexpression counteracts miR-668-5p-induced effects on osteoblast function and senescence

**Figure 9:**
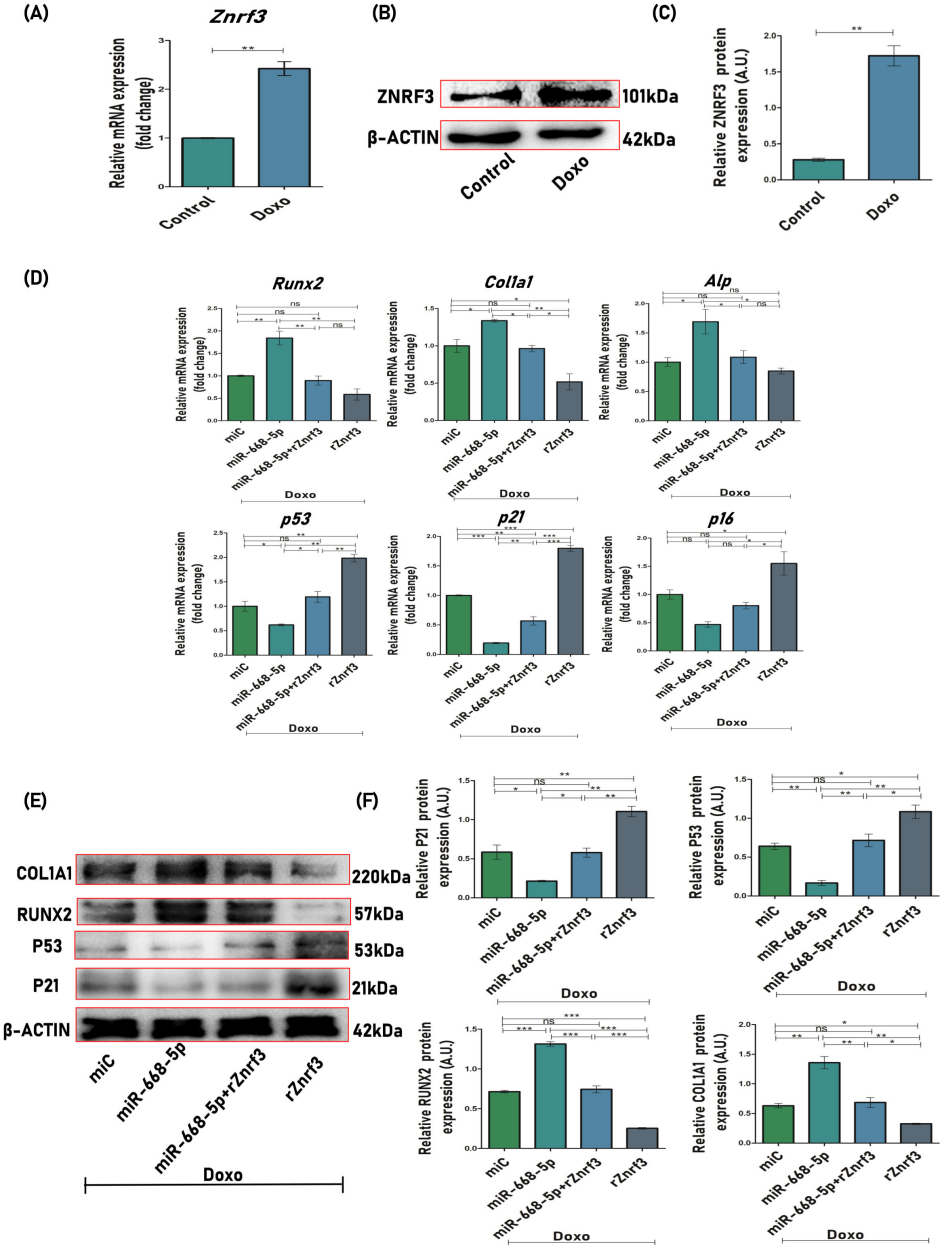
Znrf3 overexpression counteracts miR-668-5p-induced effects on osteoblast function and senescence. **(A)** qPCR analysis of Znrf3 in Doxo-treated cells (Gapdh, internal control). **(B)** Western blot of Znrf3 in Doxo-treated cells (β-Actin, internal control) and **(C)** densitometric analysis of Western blot. **(D)** qPCR analysis of osteogenic (Runx2, Col1a1 & Alp) and senescence-associated markers (p53, p21, and p16) in MCOs transfected with miR-668-5p in the presence and absence of rZnrf3 in Doxo conditions (Gapdh, internal control). **(E)** Western blots of osteogenic (Runx2 and Col1a1) and senescence-associated markers *(*p53 and p21) (β-Actin, internal control) and **(F)** densitometric analysis of Western blots in MCOs transfected with miR-668-5p in the presence and absence of rZnrf3 in Doxo conditions. Data are expressed as mean ± SEM (*n* = 3); *p < 0.05, **p < 0.01, ***p < 0.001, ns (non-significant) compared between groups.

To corroborate our previous findings, we further explored whether miR-668-5p had an influence on osteoblast differentiation and cellular senescence in a Znrf3-dependent manner. First of all, we checked the effect of Doxo on the expression levels of the miRNA target Znrf3, and it was found that Doxo-treated MCOs exhibited increased mRNA (**Figure 9A**) and protein levels of Znrf3 (**Figures 9B, C**). In Doxo conditions, osteoblasts were next transfected with miR-668-5p (mimic) in the presence or absence of 0.5 μg/ml recombinant Znrf3 (rZnrf3). qPCR and Western blot analysis interestingly revealed that miR-668-5p (mimic)–transfected cells treated with rZnrf3 (miR-668-5p+rZnrf3) exhibited increased mRNA and protein levels of p53 and p21 compared to only mimic. Although p16 mRNA levels were elevated in miR-668-5p+rZnrf3–treated cells, the change was non-significant. On the other hand, rZnrf3 treatment (with mimic) resulted in reduced mRNA levels of osteogenic markers (Runx2, Col1a1, Alp) (**Figure 9D**) and further decreased Runx2 and Col1a1 protein levels as compared to only mimic transfected cells (without rZnrf3), thereby counteracting the protective effects of miR-668-5p (**Figures 9E, F**). These results indicate that miR-668-5p targets Znrf3 to resist Doxo-induced cellular senescence and the associated suppression of osteoblast differentiation.

## 4 Discussion

In the present study, we identified what is, to our knowledge, an uncharacterized function of miR-668-5p, which promotes bone formation by suppressing cellular senescence via targeting Znrf3. Though there are no direct effects of miR-668-5p, a report has identified miR-668-3p as a potential therapeutic target for AD, an aging-related neurodegenerative disorder, by regulating oxidative stress (19). The isoform miR-668-3p has been investigated across various other conditions also, including AKI, ischemia-reperfusion injury (IRI), and osteoblast progression in osteonecrosis, where it demonstrated protective effects. The functions of miR-668-5p, however, remain to be explored, especially in bone. Interestingly, more than a three-fold reduction in levels of miR-668-5p was observed in miRNA profiling data of Phex-silenced osteoblasts, and it is reported that protein levels of Phex in the femur and calvaria, along with the mRNA levels in most tissues (except brain), decline with aging (44, 45). These findings suggest a potential link between Phex dysregulation and age-associated bone loss and have led to a growing interest in exploring the involvement of Phex in such conditions, particularly through its regulation by microRNAs. Keeping the background information in the picture, we decided to explore the role of miR-668-5p in osteoblast senescence and aging-induced bone loss conditions.

We investigated the osteoprotective effects of miR-668-5p under aging conditions utilizing both *in-vitro* and *in-vivo* approaches. For this, we comprehensively evaluated the action of miR-668-5p on osteoblastogenesis, its downstream target, signaling mechanism, role in osteoblast senescence, oxidative stress, and aging-associated bone loss. The findings of this study shed light on the intricate interplay between miRNA signaling, bone loss, and cellular senescence, providing novel insights into the molecular underpinnings of age-related skeletal decline. Our results demonstrate that the overexpression of miR-668-5p increased osteoblast differentiation, mineralization, and expression of osteogenic markers like Runx2, Col1a1, and Alp. All these effects were diminished by anti–miR-668-5p. Potential targets were identified using target prediction tools such as TargetScan and miRDB. Among the predicted targets, Znrf3, an E3 ubiquitin ligase, was of particular interest, as it is known to negatively regulate Wnt signaling, a critical pathway for osteoblastogenesis and bone formation. Prior research has shown that Znrf3 acts as a suppressor of the Wnt/β-catenin signaling cascade (38, 46). Target prediction was supported by a significant reduction in Znrf3 transcript and protein levels in miR-668-5p mimic-transfected cells. Further validation using the DLR assay demonstrated that overexpression of miR-668-5p (mimic) suppressed the luciferase activity of the Znrf3 3′ UTR reporter construct. Correspondingly, protein expression studies revealed that miR-668-5p (mimic) transfection enhanced Wnt signaling components by targeting Znrf3 and reducing its inhibitory effect on the pathway. These findings suggest that one of the potential mechanisms by which miR-668-5p facilitates osteoblast function is by promoting Wnt signaling through the downregulation of Znrf3.

Wnt signaling has been shown to decline with age, contributing to a reduction in osteoblast numbers and the onset of cellular senescence (5, 47). Stevens et al. have reported that Wnt10b deficiency results in age‐dependent loss of bone mass and progressive reduction of mesenchymal progenitor cells (48). Additionally, a study by Rauner et al. shows decreased expression of Wnt-related proteins in the bone tissue of aged mice (49). These studies point towards an association between Wnt signaling and aging. Lately, microRNAs have emerged as key regulators of cellular senescence and age-related disorders. In the context of bone homeostasis, they play a crucial role in age-related bone loss by tightly regulating cellular proliferation, differentiation, and function. They modulate key pathways involved in DNA damage, epigenetic alterations, and metabolism, which are central to aging and osteoblast senescence. For instance, miR-146a has been implicated in modulating age-related bone loss (50), while miR-138-5p targets MACF1 to exacerbate aging-induced bone loss (51). Fan et al. reported that miR-1292 regulates senescence and osteogenic differentiation via the Wnt/β-catenin pathway by targeting Fzd4 (52).

With this background, we explored the role of miR-668-5p in osteoblast senescence. Senescent cells exhibit distinct features, such as the activation of tumor suppressor pathways (e.g., p53/p21 and p16INK4a/Rb), increased β-galactosidase activity, and a pro-inflammatory secretome known as the senescence-associated secretory phenotype (SASP) (4, 53). Doxorubicin (Doxo), a commonly used chemotherapeutic agent, is well documented for its ability to induce senescence and simulate an accelerated aging phenotype (32, 54). While playing anti-tumor roles, chemotherapy drugs (like doxorubicin) induce senescence in normal tissues, contributing to cellular dysfunction (55). Moreover, therapy-induced senescence is associated with bone loss (56), and it can also spread to neighboring cells via SASP factors in a paracrine manner (57).

We standardized optimal sublethal doses for Doxo-induced osteoblast senescence at *in-vitro* and *in-vivo* levels. The levels of miR-668-5p were found to be significantly downregulated in Doxo-treated osteoblasts, and further overexpression of miR-668-5p countered Doxo-induced cellular senescence and suppression of osteoblast differentiation. On the contrary, Znrf3 expression was significantly upregulated in Doxo-treated osteoblasts, which correlates well with its inhibition of the Wnt signaling pathway. Transfection of miR-668-5p resulted in reduced Znrf3 expression in Doxo conditions as well, highlighting its target specificity. The p53-p21 pathway appeared to be significantly responsive in calvarial osteoblasts on Doxo treatment, which was targeted by miRNA overexpression. SA-β-gal activity, a widely used marker for cellular senescence (58), was also downregulated in mimic transfected osteoblast cells, highlighting its potential to prevent cellular senescence. Doxo treatment results in ROS overproduction, which becomes one of the major drivers of cellular senescence (59). Mitochondrial dysfunction, another crucial factor in aging, impairs osteogenesis and accelerates bone loss by disrupting redox balance. Doxorubicin is known to exacerbate this by targeting mitochondria form and function, elevating ROS (particularly superoxide), altering MMP, and reducing ATP production (60, 61). Our results confirmed that miR-668-5p overexpression countered ROS production, mitigated oxidative stress, and resisted Doxo-induced mitochondrial dysfunction in osteoblasts. Interestingly, a study by Gilliam et al. reports that Doxo-induced mitochondrial ROS drives skeletal muscle breakdown by increasing E3 ubiquitin ligase expression (62). Moreover, miR-668-5p also significantly upregulated Sod2 expression, suggesting a protective antioxidant mechanism.

After confirming the osteogenic and anti-senescence effect of miR-668-5p in MCOs, the same was evaluated in a Doxo-administered accelerated aging mouse model. Doxo-treated mice had significantly compromised femur bone and L5 vertebrae trabecular microarchitecture. The bone strength parameters were also compromised. miR-668-5p administration improved the trabecular deterioration caused by Doxo. In fact, it also enhanced new bone formation, promoted *ex-vivo* bone mineralization, and improved the biomechanical strength of the femur in Doxo-treated animals. These results were corroborated by evaluating serum bone markers, where miR-668-5p remarkably reduced serum CTx levels and increased serum P1NP levels, which are established bone resorption & formation markers, respectively. In addition to lowering serum CTX levels, miR-668-5p significantly reduced osteoclast number and decreased TRAP mRNA expression in mouse femurs. This indicates the potential of miR-668-5p not only as a bone-forming agent but also as an anti-resorptive entity. However, more studies are required to check the effect of miR-668-5p on osteoclasts and further explore its anti-resorptive potential. SOD activity was also significantly restored in serum samples of mimic-administered animals, suggestive of antioxidant capacity. The balance between mesenchymal stem cells (MSCs) differentiating into adipocytes or osteoblasts is controlled by key transcription factors, including PPARγ and C/EBPα, which promote adipogenesis, and RUNX2, which drives osteogenesis (43). This balance becomes even more crucial in the context of senescence-driven aging bone loss. The moderate dip observed in levels of adipogenic regulators (PPARγ and C/EBPα) and restoration of Runx2 indicate potential involvement of miR-668-5p in regulating this balance, thereby countering Doxo-induced age-related bone loss. However, given the complexity and broad scope of age-induced adipogenesis, further focused studies are necessary to fully elucidate the specific role of miR-668-5p in this context.

Research by Yu et al. indicates that p53 plays a critical role in the pathogenesis of osteoporosis (63). Furthermore, the targeted removal of p21-positive senescent cells has been demonstrated to prevent osteoporosis induced by radiation (64). In the Doxo+miC group, the expression levels of senescence markers such as p53 and p21 were significantly elevated, whereas treatment with miR-668-5p reduced their expression. Conversely, Doxo was found to suppress osteogenesis by downregulating Runx2, the key transcription factor for osteoblast differentiation, and Type I collagen (Col1a1) expression, while miR-668-5p administration restored their expression.

As miR-668-5p predominantly exerted its senescence-mitigating effect via targeting Znrf3, a Wnt signaling inhibitor, it was pertinent to understand whether replenishing Znrf3 in mimic transfected cells would reverse the anti-aging effects of miR-668-5p. In our study, the administration of exogenous Znrf3 counteracted the effects of miR-668-5p on Doxo-treated osteoblasts, confirming the target specificity of miR-668-5p. Increased expression of senescence markers with a concomitant decrease in osteogenic marker expression was observed in mimic transfected osteoblasts supplemented with recombinant Znrf3 in Doxo conditions. According to a recent patent, the inhibition of Znrf3 has been proposed as a potential strategy for treating decreased bone mineral density (65). Our findings are instrumental in depicting a key role of Znrf3 in osteoblast senescence. However, additional studies are warranted to validate this hypothesis.

Our study, thus, highlights the role of miR-668-5p as a potential biomarker and therapeutic target in age-related bone degeneration exacerbated by chemotherapeutic interventions. Although our study was conducted in preclinical models, the findings have potential clinical relevance. Doxorubicin-induced bone loss and skeletal fragility are emerging complications in cancer survivors, and current therapeutic options are limited (23). By identifying miR-668-5p as a regulator of osteoblast function that targets ZNRF3 to activate Wnt/beta-catenin signaling, our study provides a mechanistic basis for considering miR-668-5p in the development of novel strategies to mitigate chemotherapy-associated and age-related bone deterioration. Future work using human bone tissue or patient-derived samples will be critical to translate these findings into clinical settings. Moreover, further studies are needed to validate these findings and address existing limitations, as miRNA therapeutics often face challenges like off-target effects and instability, necessitating advanced delivery systems such as nanocarriers, exosomes, and lipid nanoparticles for improved precision and biocompatibility.

**Figure 10:**
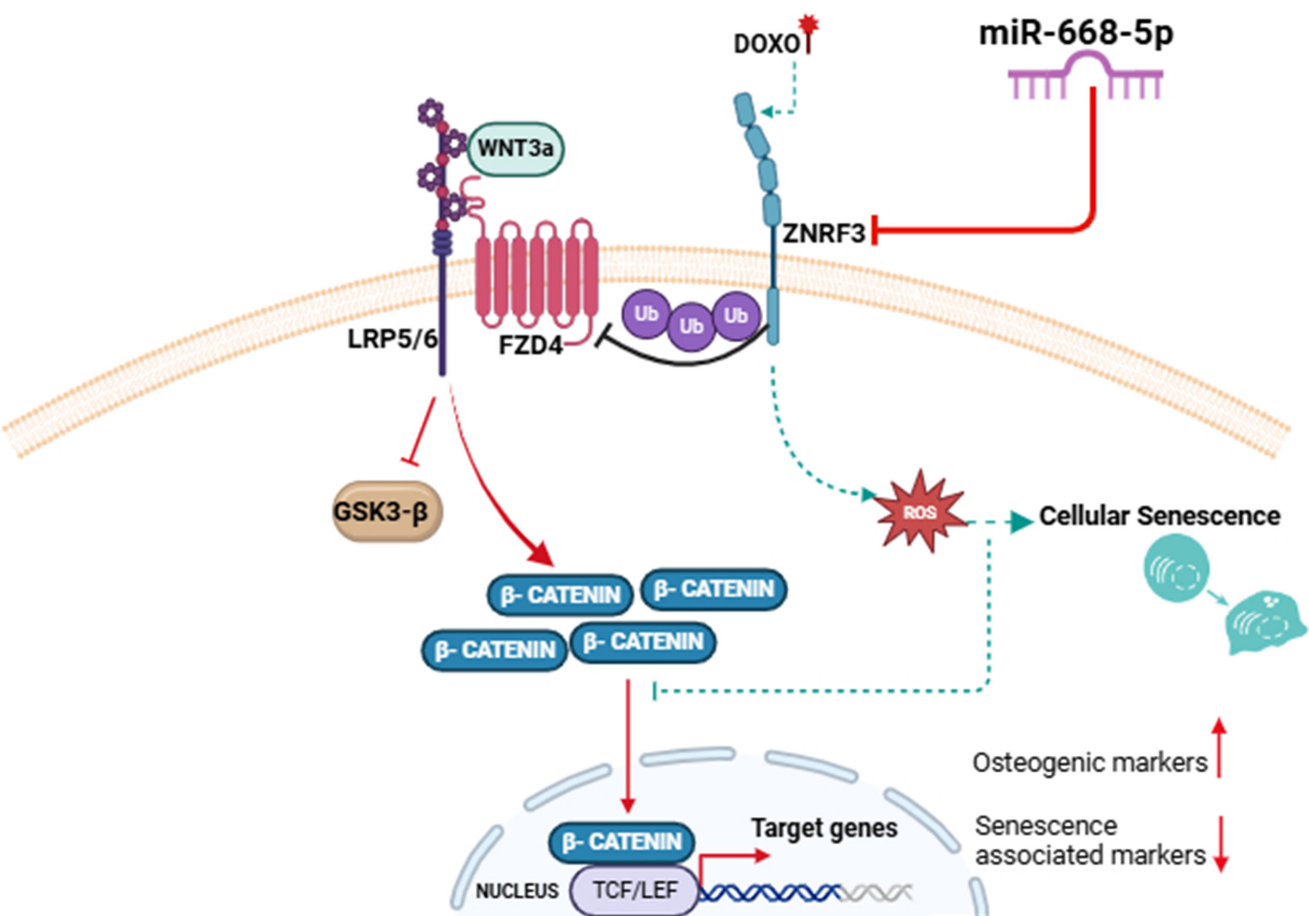
Graphical abstract—MiR-668-5p targets ZNRF3, upregulates Wnt/β catenin signaling pathway in osteoblasts, and mitigates Doxorubicin-induced aging bone loss.

In summary (**Figure 10**), our study demonstrates that Doxo-induced aging bone loss associated with osteoblast senescence is alleviated by a novel miRNA candidate, miR-668-5p, by targeting an E3 ubiquitin ligase Znrf3, and associated Wnt signaling. Overexpression of miR-668-5p upregulates osteoblast functions while significantly mitigating cellular senescence and oxidative stress. Thus, the current study provides compelling evidence for the potential of miR-668-5p as an osteoprotective agent under aging conditions.

## Data Availability

The original contributions presented in the study are included in the article/**Supplementary Material**. Further inquiries can be directed to the corresponding author.
