# Supplementary material for: MiR-668-5p targets ZNRF3, an E3 ubiquitin ligase to enhance osteoblast function and alleviate senescence in doxorubicin-induced age-related bone loss
Source: Front Endocrinol (Lausanne). 2025 Oct 27;16:1693659. doi: 10.3389/fendo.2025.1693659 (PMC12597718; doi:10.3389/fendo.2025.1693659)
Supplement: Supplementary file 1 [file DataSheet1.docx]

## **SUPPLEMENTARY INFORMATION**

## **MiR-668-5p targets ZNRF3, an E3 ubiquitin ligase to enhance osteoblast function and alleviate senescence in doxorubicin-induced age-related bone loss**

Devendra Pratap Singh1,2, Alok Tripathi1,2, Ankita Paul1, Saurabh Kumar Kaushal1,2, Megha Dixit1,2, Divya Singh1,2,*

*1Division of Endocrinology, CSIR-Central Drug Research Institute, Lucknow 226031, U.P., India*

*2Academy of Scientific and Innovative Research (AcSIR), Ghaziabad- 201002, India.*

*** Corresponding Author** Dr. Divya Singh, Chief Scientist, Division of Endocrinology, CSIR-Central Drug Research Institute, Lucknow, 226031, U.P., India.

E-mail: divya_singh@cdri.res.in

divya.singh.cdri@csir.res.in

**SUPPLEMENTARY INFORMATION**

**1 miRNA description**

[*Mus musculus*](https://www.mirbase.org/results/?query=mmu)- mmu-miR-668-5p

Accession no. [MIMAT0017237](https://mirbase.org/mature/MIMAT0017237)

Mature miRNA sequence (5’-3’) **GUAAGUGUGCCUCGGGUGAGCAUG**

**2 MATERIAL & METHODS**

**2.1 Cell culture (MCO culture)**

The head of the neonatal mouse pups was sterilized with 70% ethanol and decapitated. Under sterile conditions, the calvariae were excised and further cleaned of connective tissue using fine instruments before being transferred to sterile PBS. Harvested calvariae were further subjected to five sequential digestions at 37°C using a solution containing 0.1% dispase (Gibco) and 0.1% collagenase (Gibco). Cells obtained from the second to fifth digestion cycles were collected, pooled, and centrifuged. The resulting cell pellet was resuspended in alpha-MEM (Sigma) medium and plated in T25 flasks containing complete growth medium i.e. alpha-MEM supplemented with 10% FBS (Gibco) and 1% penicillin/streptomycin (Sigma). For treatment purposes, cells were maintained in osteoblast differentiation medium containing alpha-MEM with 5% FBS, 10 mM β-glycerophosphate (Sigma), 50 μg/mL ascorbic acid (Sigma), and 1% penicillin/streptomycin.

**2.2 Immunofluorescence staining**

For immunofluorescence, ZNRF3 antibody (Affinity Biosciences, DF14289) at a dilution of 1:250 was used and was carried out following previously published standard protocol (1).

**2.3 Measurement of Mitochondrial ROS**

To evaluate mitochondrial ROS, particularly superoxide, MitoSOX Red dye (Invitrogen) (5µM) was used to stain the cells for 15-20 minutes at 37°C under dark conditions. After washing with 1x PBS, imaging was carried out using a fluorescence microscope (20x) (EVOS) aided with appropriate filters (2,3). Mitochondrial membrane potential (MMP) was evaluated using Rhodamine 123 fluorescent dye (Sigma) (10μg/mL in PBS) in a 96-well plate format. After treatment, cells were stained with 10μL of Rhodamine 123 dye for 30 minutes at 37°C. After incubation, cells were washed with 1x PBS twice. Fluorescence intensity was measured using a plate reader at Ex/Em=485/530 nm to assess MMP (2). For ATP measurement, an ATP assay kit (colorimetric) (Abcam, ab83355) was used following the manufacturer’s instructions.

**2.4 Cell Apoptosis Assay using Annexin V-FITC/PI staining**

FITC Annexin V Apoptosis Detection Kit (BD Biosciences, 556547) was used to quantitatively determine % of cells that were actively undergoing apoptosis following the manufacturer’s protocol. The treated cells were harvested, washed twice with PBS, and resuspended in 100 µL of Annexin V binding buffer. Annexin V-FITC (5 µL) and propidium iodide (PI, 5 µL) were added, and the mixture was incubated in the dark at room temperature for 15 minutes. After incubation, 400 µL of Annexin V binding buffer was added, and the samples were analyzed using flow cytometry (BD FACS Calibur).

**2.5 Body Composition Analysis**

The body weight and body composition of animals of respective groups were analyzed a day before autopsy with the help of Echo-MRI Body Composition Analyzer E26-226-RM (Echo MRI LLC), following a previously published protocol (1).

**Supplementary Table 1.** List of Primers used for qPCR analysis

| **Gene** | **Forward primer** (5’ - 3’) | **Reverse primer** (5’ - 3’) |
| --- | --- | --- |
| ***Gapdh*** | GGAGCGAGATCCCTCCAAAAT | GGCTGTTGTCATACTTCTCATGG |
| ***Znrf3*** | AGGAAGAGTTTGGCCGAGTG | ATGGCCACTTCCTAAAGGGC |
| ***Col1a1*** | AAGAGAGAGGCAGAGGGGAG | CCCTGCCCACTCTCTACTCT |
| ***Alp*** | GGGCAATGAGGTCACATCCA | GTCGGTTCTGTTCTTCGGGT |
| ***Runx2*** | CGCCTCACAAACAACCACAG | AGCACTCACTGACTCGGTTG |
| ***p53*** | GTGCTCACCCTGGCTAAAGT | TGGGAAGGAGGAGGATGAGG |
| ***p21*** | CACCGAGCCTGTTTCTCTGT | CCACAGCACAGGAGTCACAT |
| ***p16*** | AGGACCCCACTACCTTCTCC | CAGCGGAACACAAAGAGCAC |
| ***Sod2*** | TGCTGGGATTAAAGGCGTGT | CAGCAAACACAGAGTGGCAC |
| ***Bax*** | GTGAGCGGCTGCTTGTCT | GGTCCCGAAGTAGGAGAGGA |
| ***Bcl2*** | GTACCTGAACCGGCATCTG | GGGGCCATATAGTTCCACAA |
| ***Trap*** | GCGACCATTGTTAGCCACATACG | CGTTGATGTCGCACAGAGGGAT |
| ***C/ebp-α*** | GCAAAGCCAAGAAGTCGGTGGA | CCTTCTGTTGCGTCTCCACGTT |
| ***Ppar-γ*** | GTACTGTCGGTTTCAGAAGTGCC | ATCTCCGCCAACAGCTTCTCCT |

**Supplementary Table 2 -** List of Antibodies used for Western Blotting

| **Antibodies** | **Company, Catalog no. (dilution)** |
| --- | --- |
| RUNX2 | Affinity, AF5186 (1:1000); Abclonal, A2851 (1:1000) |
| COL1A1 | CST, 72026 (1:1000) |
| ALP | Proteintech, 11187-1-AP (1:1000) |
| ZNRF3 | Affinity, DF14289 (1:1000) |
| FZD4 | Affinity, DF13439 (1:1000) |
| LRP6 | CST, 3395 (1:1000) |
| WNT3A | Proteintech, 26744-1-AP (1:1000) |
| GSK3β | CST, 9315 (1:1000) |
| β-CATENIN | CST, 9562 (1:1000) |
| PHOSPHO-β-CATENIN | CST, 9561 (1:1000) |
| P53 | CST, 2524 (1:1000) |
| P21 | Abcam ab109199 (1:1000) |
| P16 | Invitrogen PA5-20379 (1:1000) |
| SOD2 | CST, 13194 (1:1000) |
| BAX | Affinity, AF0120 (1:1000) |
| BCL2 | Affinity, AF6139 (1:1000) |
| β-ACTIN | CST, 4970S (1:1000); Sigma, A3854 (1:25000) |
| HRP CONJUGATED ANTI-RABBIT | Jackson Immuno, 111-035-003 (1:5000) |
| HRP CONJUGATED ANTI-MOUSE | Jackson Immuno, 115-035-003 (1:5000) |

**3 Results**

**
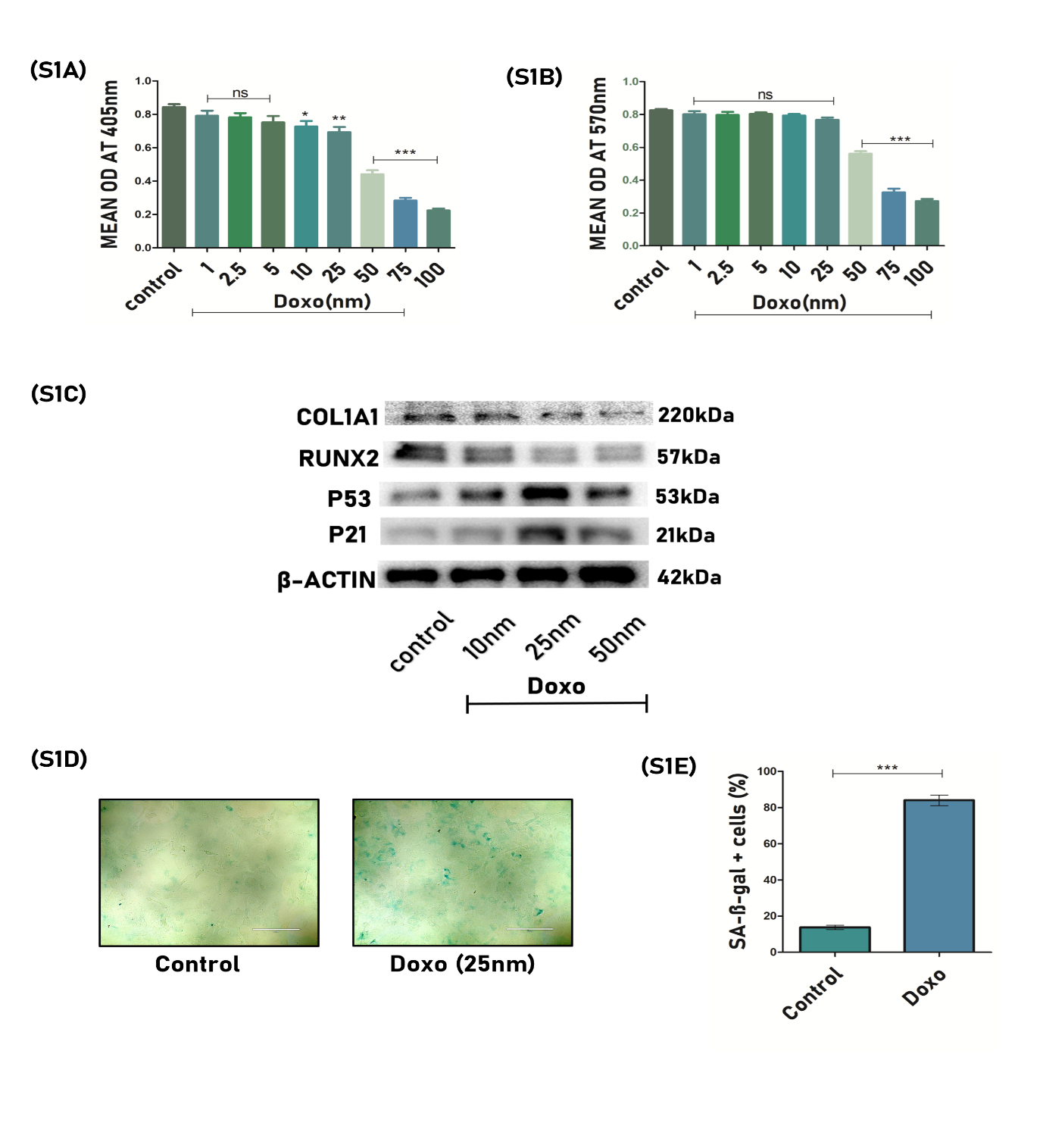
Supplementary Figure 1. Doxorubicin dose standardization to induce cellular senescence in MCOs**. **(A)** ALP & **(B)** MTT assay of MCOs treated with different concentrations of Doxorubicin (Doxo). **(C)** Western Blots of osteogenic (Runx2 & Col1a1) and senescence-associated markers (p53 & p21) in MCOs treated with different concentrations of Doxo. **(D)** Representative images (20x) of SA-β-gal (Senescence-associated β-galactosidase) staining **(E)** SA-β-gal-positive cells (%) quantification. Data are expressed as mean ± SEM (n=3); *p < 0.05, **p < 0.01, ***p < 0.001, compared between groups and w.r.t. control in ALP & MTT assay, ns (non-significant).


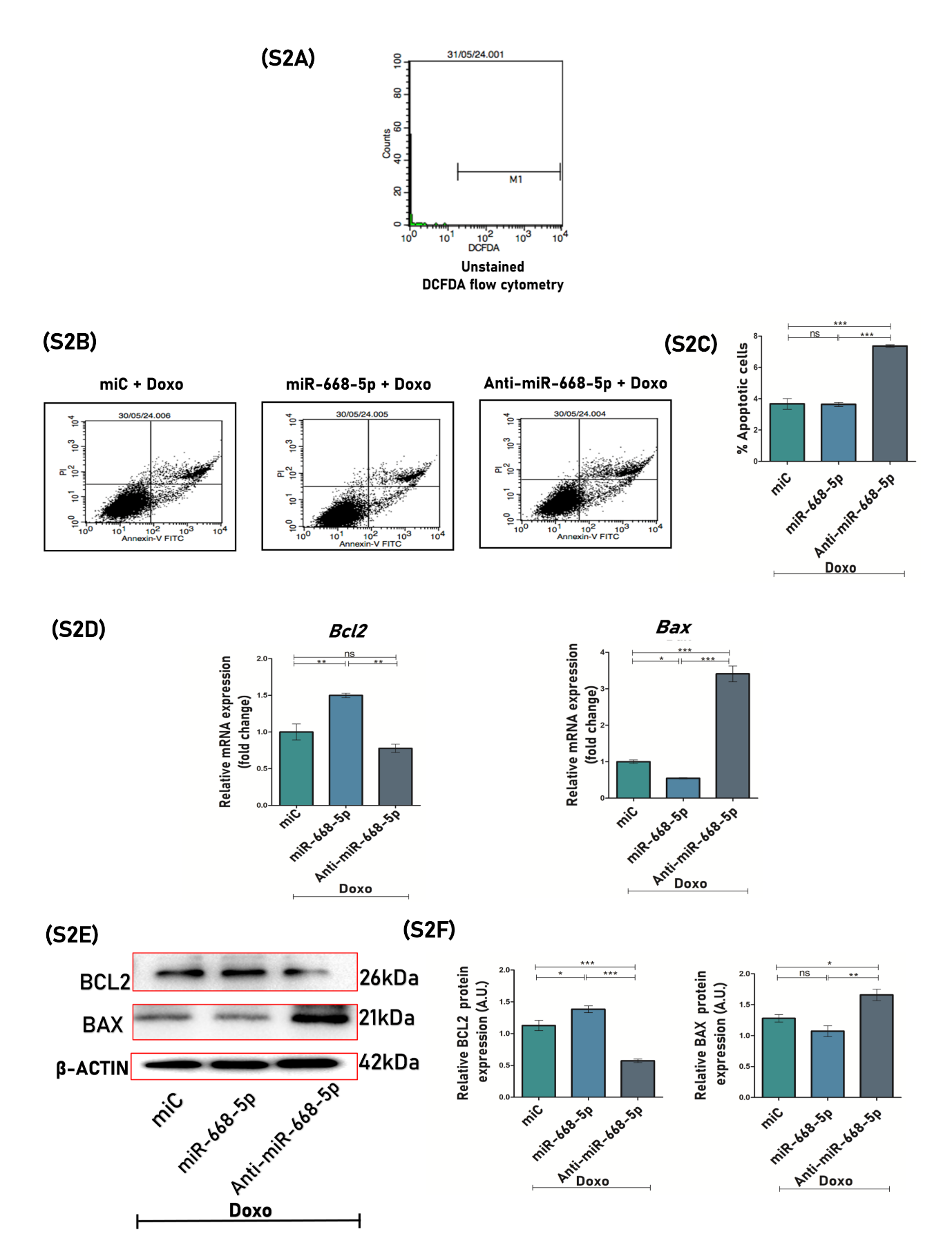


**Supplementary Figure 2. miR-668-5p overexpression counters Doxorubicin-induced ROS production and mitochondrial dysfunction. (A)** Unstained cells for DCFDA ROS flow cytometry assay **(B)** Annexin PI staining using flow cytometry and **(C)** quantification of % Apoptotic cells. **(D)** qPCR analysis (Gapdh, internal control), **(E)** Western Blots of apoptotic regulators Bcl2 & Bax in miR transfected MCOs in presence of Doxo (β-actin, internal control), and **(F)** densitometric analysis of Western blots. Data are expressed as mean ± SEM (n=3); *p < 0.05, **p < 0.01, ***p < 0.001, ns (non-significant) compared between groups.

**
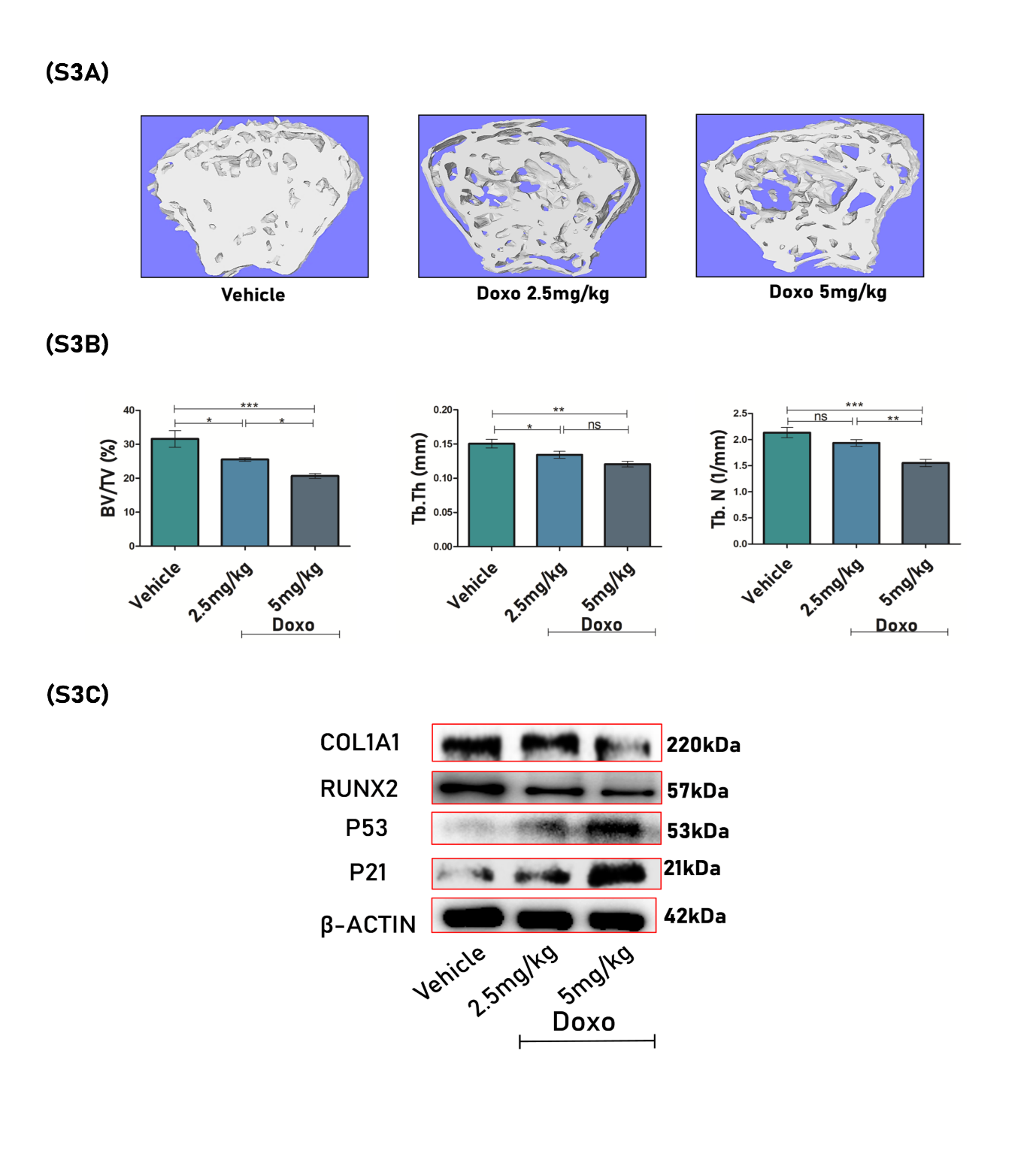
**

**Supplementary Figure 3. Doxorubicin dose standardization for animal study**. **(A)** 3D micro-CT representations of mice femur trabecular network exposed to two different Doxorubicin doses 2.5mg/kg and 5mg/kg. **(B)** Quantification of trabecular microarchitecture parameters (BV/TV%, Tb.N., & Tb.Th.). **(C)** Western Blots of osteogenic (Runx2 & Col1a1) and senescence-associated markers (p53 & p21) in mice femur bone samples. Data are expressed as mean ± SEM (n=6); *p < 0.05, **p < 0.01, ***p < 0.001, ns (non-significant) compared between groups.

**Abbreviations** MCOs, Mouse calvarial osteoblasts; Runx2, Runt-related transcription factor 2; Col1a1, collagen type I alpha 1 chain; Alp, Alkaline phosphatase; Phex, Phosphate-Regulating Endopeptidase X-Linked; Doxo, Doxorubicin; Znrf3, Zinc and ring finger 3; ARS, Alizarin Red Staining; DLR; Dual Luciferase Reporter; SA-β-gal, Senescence-associated β -galactosidase**;** ROS, Reactive oxygen species; Micro-CT, Micro-computed tomography; BV/TV, Bone volume per tissue volume; Tb.Sp., Trabecular separation; Tb.N., Trabecular number; Tb.Th. Trabecular thickness; BS/TV, Bone surface density; SMI, Structure model index; Tb.Pf., Trabecular bone pattern factor; BMD, Bone mineral density; MS/BS, Mineralizing surface; MAR, Mineral apposition rate; BFR, Bone formation rate; P1NP, Procollagen type 1 N-terminal propeptide; CTx, C-terminal telopeptide of type I collagen; SOD, Superoxide dismutase. TRAP, Tartrate-Resistant Acid Phosphatase; C/EBPα, CCAAT/enhancer-binding protein alpha; PPARγ, Peroxisome Proliferator-Activated Receptor Gamma.

**References**

1. Rai R, Singh KB, Khanka S, Maurya R, Singh D. Cladrin alleviates dexamethasone-induced apoptosis of osteoblasts and promotes bone formation through autophagy induction via AMPK/mTOR signaling. *Free Radic Biol Med* (2022) 190:339–350. doi: 10.1016/j.freeradbiomed.2022.08.028

2. Sharma K, Kumar S, Prakash R, Khanka S, Mishra T, Rathur R, Biswas A, Verma SK, Bhatta RS, Narender T, et al. Chebulinic acid alleviates LPS-induced inflammatory bone loss by targeting the crosstalk between reactive oxygen species/NFκB signaling in osteoblast cells. *Free Radic Biol Med* (2023) 194:99–113. doi: 10.1016/j.freeradbiomed.2022.11.026

3. Sharma A, Singh S, Ahmad S, Gulzar F, Schertzer JD, Tamrakar AK. NOD1 activation induces oxidative stress via NOX1/4 in adipocytes. *Free Radic Biol Med* (2021) 162:118–128. doi: 10.1016/j.freeradbiomed.2020.11.036
